# Using the grip strength effort task to measure reward processing dysfunction in schizophrenia and major depressive disorder

**DOI:** 10.1016/j.nsa.2025.105524

**Published:** 2025-07-30

**Authors:** Asad Malik, Shannon N. Millard, Ailidh Finlayson, Amy Beckenstrom, Abigail Abrahams, Dennis Hernaus, Stella D. Voulgaropoulou, Therese van Amelsvoort, Daniel Umbricht, William J. Martin, Darrel J. Pemberton, Jane Tiller, Anke Sambeth, Oliver Grimm, Michael M. Plichta, Andreas Reif, Charis Styliadis, Athanasia Liozidou, Georgios Papazisis, Victor Pérez, Matilde Elices, Karla V. Allebrandt, Stephane Pollentier, Gerard R. Dawson

**Affiliations:** a P1vital Products Ltd., Wallingford, United Kingdom; b Department of Psychiatry and Neuropsychology, Mental Health and Neuroscience (MHeNs) Research Institute, Maastricht University, Maastricht, Netherlands; c Xperimed LLC, Basel, Switzerland; d Johnson & Johnson Innovative Medicine, Beerse, Belgium; e Faculty of Psychology and Neuroscience, Maastricht University, Maastricht, Netherlands; f Department of Psychiatry, Psychosomatic Medicine and Psychotherapy, University Hospital Frankfurt, Frankfurt, Germany; g Medical Physics and Digital Innovation Laboratory, Aristotle University of Thessaloniki, Thessaloniki, Greece; h Laboratory of Cognitive Neuroscience and Clinical Neuropsychology, The Scientific College of Greece, Athens, Greece; i Department of Clinical Pharmacology, School of Medicine, Aristotle University of Thessaloniki, Thessaloniki, Greece; j Instituto de Salud Mental, Hospital del Mar, Universitat Pompeu Fabra, Barcelona, Spain; k Hospital del Mar Research Institute (IMIM), Barcelona, Spain; l Centro de Investigación Biomédica en Red de Salud Mental (CIBERSAM), Madrid, Spain; m Translational Medicine and Clinical Pharmacology, Boehringer Ingelheim Pharma GmbH & Co. KG, Germany; n School of Medicine, European University Cyprus, Frankfurt, Germany; o Medicine, Boehringer Ingelheim International GmbH, Binger Straße 173, 55216 Ingelheim am Rhein, Germany

**Keywords:** Schizophrenia, Depression, Anhedonia, Apathy, Reward processing, Effort-based decision-making task, Negative symptoms

## Abstract

The aims of the Reward Task Optimisation Consortium (RTOC) study (Bilderbeck et al., 2020) were to explore the validity, reliability, and feasibility of a battery of reward processing tasks for the development of new treatments for anhedonia. We report our findings from the Grip Strength Effort Task (GSET), an effort-based decision-making task in which participants chose to either perform easy trials that required less physical effort for low monetary reward or hard trials that required more effort for potentially larger rewards. Thirty-seven participants with schizophrenia (SZ), 40 with major depressive disorder (MDD), and 59 age- and sex-matched healthy controls were administered the task across four European sites. 19% of participants (8.5% HC, 27% SZ, 27.5% MDD) were ‘inflexible responders’ who always chose hard trials irrespective of the reward amount. MDD participants showed less willingness to exert physical effort for high reward than controls, when inflexible responders were excluded, but no statistically significant differences were observed between SZ participants and controls. Across all participants, willingness to exert effort for high reward negatively correlated with measures of anhedonia. Inflexible responders exhibited higher depressive symptoms and higher anticipation of punishment than other participants. Forty-three participants performed the GSET again after 3–5 weeks and moderate-to-high test-retest reliability was observed. Minimal site effects confirmed operational feasibility of the task in multi-site studies. We conclude that the GSET can provide objective behavioural biomarkers of reward processing dysfunction, but further investigation is needed to understand inflexible responding and its implications on the task's design and interpretation.

## Highlights

• Grip Strength Effort Task was used to measure anhedonia through effort for reward.
• Unexpectedly, 19 % participants always chose hard trials, regardless of reward amount.
• Excluding inflex. responders, MDD chose fewer high-reward hard trials than controls.
• SZ showed no significant differences from controls.
• Willingness to exert effort negatively correlated with symptoms of anhedonia.

## 1 Introduction

Anhedonia, a loss of interest in and diminished ability to experience pleasure, is a symptom observed in many psychiatric disorders, including major depressive disorder (MDD (Loas, 1996);) and schizophrenia (SZ (Andreasen and Olsen, 1982);). In clinical practice, anhedonia is closely linked to impaired motivation, loss of interest, apathy, and social withdrawal. These clinical characteristics predict poor functional outcomes for patients (McMakin et al., 2012), particularly social functioning (Grove et al., 2016; Kiwanuka et al., 2014; Kupferberg et al., 2016), and have significant effects on quality of life (Fervaha et al., 2014). There are currently no approved treatments for anhedonia, despite its high prevalence across a wide range of psychiatric indications. One way to address this unmet clinical need is to advance our understanding of the components that drive anhedonia, which can be done by investigating assumed underlying neuropsychological and neurobiological mechanisms, such as reward valuation, effort-allocation/motivation, anticipation, and consummatory pleasure (Gorwood, 2008; Saleh et al., 2021; Treadway and Zald, 2013).

The Reward Task Optimisation Consortium (RTOC (Bilderbeck et al., 2020);) was formed by a group of industry and academic partners initially supported by the European College of Neuropharmacology (ECNP) to explore the sensitivity and feasibility of three reward processing tasks for use in the development of new treatments for anhedonia – the Grip Strength Effort Task (GSET (Reddy et al., 2015);), the Doors Task (Foti and Hajcak, 2009), and the Working Memory/Reinforcement Learning Task (Collins et al., 2017). The Research Domain Criteria (RDoC), a transdiagnostic framework for understanding mental health disorders in terms of neurobiological and behavioural dimensions (Cuthbert and Insel, 2013), informed the study design. The tasks in the RTOC battery were selected based on their ability to probe specific RDoC (sub)domains of learning, effort valuation, and reward processing (Whitton et al., 2015). Participants with schizophrenia, major depressive disorder, and healthy controls (HC), were recruited and administered these tasks, along with several clinical questionnaire-based scales, with the goal of developing a valid, reliable, neuroscience-informed digital test battery for the measurement of anhedonia and dysfunctional reward processing. The study was implemented across four sites in Germany, Greece, the Netherlands, and Spain.

Effort-based decision-making tasks have emerged as a way to examine reward dysfunction in animal and human populations, through assessing how much effort a participant is willing to exert for a specified reward (Costello et al., 2024; Whitton et al., 2015). Effort-based decision-making tasks usually require participants to choose between easy or hard (physically or cognitively) trials for varying amounts of reward (e.g., Reddy et al. (2015); Treadway et al. (2009)). The decision to expend greater effort for larger rewards hinges on factors related to reward processing, such as the perceived effort required to successfully complete the task, the subjective valuation of the potential reward, motivation to exert effort, and the probability of receiving reward upon successful completion. Effort-based decision-making tasks can, therefore, potentially provide objective measures and biomarkers of these domains of reward processing, which may be sensitive to drug treatments that aim to modulate the neural circuitry underlying reward-related behaviours. Several recent studies have explored the transdiagnostic aspects of both physical and cognitive effort-based decision-making in psychotic and mood disorders (Barch et al., 2023; Culbreth et al., 2024; Moran et al., 2023; Zou et al., 2020). All of these studies found that SZ participants, exhibited reduced willingness to exert effort for rewards than HCs, especially at high reward, but only Zou et al. (2020) observed this in MDD. Notably, task measures were also associated with other symptomatic measures, such as pleasure and motivation, working memory, and engagement in goal-directed activities (Barch et al., 2023; Moran et al., 2023).

The Grip Strength Effort Task (GSET) is a computerised effort-based decision-making task that measures how an individual's willingness to exert greater physical effort is modulated by the magnitude of the reward on offer. Variants of this task have shown differences in willingness to exert effort for reward between controls and SZ participants (Hartmann et al., 2015; Reddy et al., 2015) and MDD (Cathomas et al., 2021; Cléry-Melin et al., 2011), although to date it has not been explored transdiagnostically in these populations. It has also shown sensitivity to drug-induced improvements in performance in healthy individuals (Wardle et al., 2011). This task was included in RTOC to further assess its suitability in clinical trials in terms of case-control differences, associations with clinical scales, replications of previous findings, test-retest reliability, and operational feasibility across multiple, international, sites. In RTOC we aimed to include populations with different psychiatric disorders to follow a more transdiagnostic approach, in line with the RDoC framework. Findings on the test-retest reliability of the GSET are currently limited (but see Reddy et al. (2015)) for a comprehensive assessment of the reliability of several effort-based decision-making tasks) and often highly specific (i.e. addressing a specific task in the context of a specific indication), which tends to limit the ability to compare and contrast the results across treatment classes and distinct clinical groups. Here, we report our findings on the behavioural data from the GSET to shed further light on the extent to which its outcomes can be considered clinically meaningful objective biomarkers of reward processing dysfunction for use in clinical trials.

## 2 Materials and methods

### 2.1 Participants

The RTOC study was conducted at four European research centres spanning the Netherlands (Maastricht University Medical Centre), Germany (University Hospital Frankfurt), Spain (Institute of Neuropsychiatry and Addictions, Barcelona), and Greece (Aristotle University of Thessaloniki). Participants were recruited via: (i) existing participant networks, (ii) clinics at each study site and (iii) through various media such as newspapers, posters, flyers, radio, mail-lists, and social media. Thirty-nine individuals with a diagnosis of schizophrenia (SZ), 41 with a diagnosis of major depressive disorder (MDD), and 60 age- and sex-matched healthy controls (HC) were recruited. Three participants were later withdrawn as they did not meet the enrolment criteria: one case of alcohol use disorder (MDD) and two cases of disallowed concomitant medication (both SZ). All participants were aged between 20 and 55 years, inclusive. CNS medications were permitted in the SZ and MDD groups provided the medication, or its daily dose, had not been changed by more than ± 30 % within 4 weeks of starting the study and was not expected to change by a larger fraction while participating in the study. Briefly, healthy control selection criteria included no current nor prior Diagnostic and Statistical Manual of Mental Disorders 5th edition (DSM-5) diagnosis of MDD or any psychotic or schizoaffective disorder, Quick Inventory of Depressive Symptomatology (QIDS-SR16) score of ≤5, and no current nor prior prescription of any psychotropic medication. MDD participants were required to have a primary DSM-5 diagnosis of MDD, confirmed by the Mini-International Neuropsychiatric Interview (MINI) at screening, and meet criteria for a current Major Depressive Episode not having lasted longer than 6 months. SZ participants were required to have a primary DSM-5 diagnosis of schizophrenia, confirmed by the MINI at screening. Further details on the inclusion/exclusion criteria are provided in the associated protocol manuscript (Bilderbeck et al., 2020). For analysis, as pre-specified in the protocol manuscript, the healthy-controls were split into overlapping MDD-HC and SZ-HC sets, that were matched to their corresponding patient groups in age and sex (see Supplementary Material for details).

### 2.2 Study design

Participants performed a baseline visit consisting of screening, completion of three reward processing tasks, and several clinical assessments and questionnaires sensitive to anhedonia, avolition, and other negative symptoms. All self-report questionnaires and behavioural tasks were delivered in a computerized format through the ePRO system developed by P1vital Products Limited in the sites. The task battery included the Doors (or Gambling) task (Foti and Hajcak, 2009), the Working Memory/Reinforcement Learning task (Collins et al., 2017), and the Grip Strength Effort Task (GSET (Reddy et al., 2015);). The GSET and Doors task were performed in conjunction with EEG. Further details of the protocol are described in Bilderbeck et al. (2020). This manuscript focuses on the analysis and results of the behavioural data from the GSET.

As per the study protocol, 53 participants (14 SZ, 17 MDD, 22 HC) completed a retest visit, consisting of the task battery and clinical scales, a minimum of 3 weeks and a maximum of 14 weeks after baseline, adapted from an initial timeframe of 3–5 weeks due to the constraints placed on research site visits by the COVID-19 pandemic that evolved during the study. A subset of these participants (3 SZ, 3 MDD, 5 HC) performed the retest assessment online remotely, rather than in clinic visits, as in-person visits were suspended during the pandemic. The GSET and EEG recording were not performed remotely due to equipment limitations. Therefore, the GSET retest assessment was completed by 43 participants (11 SZ, 14 MDD, 17 HC, median days after baseline = 28, IQR = 23 to 32). At retest, if there was a recent change in medication or a new medical event such that the participant no longer met the inclusion criteria, they were excluded from retest data collection.

### 2.3 Grip Strength Effort Task (GSET)

**Fig. 1:**
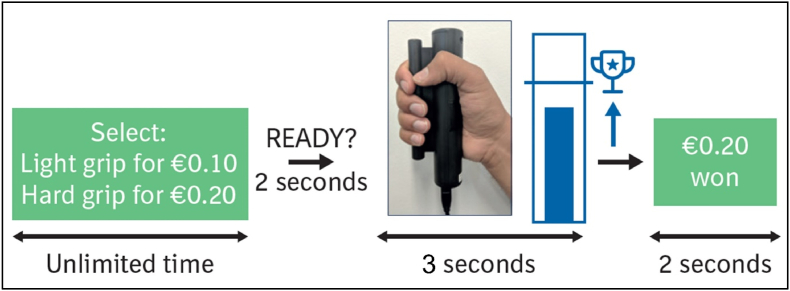
Example of a single trial. Medium reward was offered for the hard trial choice alongside the fixed small reward for the easy trial choice. The hard choice was selected, and the target grip strength was achieved, thereby winning the medium reward. Adapted from Bilderbeck et al. (2020) under the Creative Commons Attribution License (CC BY).

We implemented the GSET as described in Reddy et al. (2015). Across 54 trials, participants were required to squeeze a hand grip device (HHSC-1 × 1-GRFC; Current Designs, Inc.) to earn monetary rewards (Fig. 1). On each trial, participants chose between an easy trial (light grip; 50 % maximum grip force) or the hard trial (hard grip; 90 % maximum grip force). Grip force levels were based on a pre-task calibration session during which participants’ average maximum grip strength was measured (twice with each hand). The reward for successfully completing an easy grip trial was always a specific lower amount (€0.10) whereas the reward associated with hard trials varied equally across three levels (€0.10, €0.20 or €0.40). Participants used their dominant hand to perform the task and were given real-time visual feedback via a digital “thermometer” scale marked with a target line. They received the reward as soon as they reached the target, and did not receive it if they failed to so within 3 s. Before the assessment phase, participants completed four practice trials containing at least one easy and one hard trial to gain familiarity with the task and to ensure they understood the instructions. Participants were informed that they would receive payment for their winnings in the task, however, in reality they received a fixed amount which was roughly the maximum that they could theoretically have won. This was explained in a debriefing session at the end of the study.

The GSET outcome measures analysed were the percentage of hard choices at each reward level and the difference between the percentage of hard choices at high reward (€0.40) and low reward (€0.10). We hypothesised that SZ and MDD participants would have lower percentages of hard choices than controls, especially for high reward, and less change in their percentage of hard choices between high and low reward compared to controls.

### 2.4 Questionnaires

Three self-report questionnaires were completed by all participants. The Snaith-Hamilton Pleasure Scale (SHAPS) provided a measure of hedonic capacity (Snaith et al., 1995), the QIDS-SR16 provided a measure of depressive symptoms (Rush et al., 2003), and the Behavioural Inhibition System/Behavioural Approach System scales (BIS/BAS) provided a measure of drive towards a desired goal or stimulus (BAS) and of desire to avoid an unpleasant situation or outcome (BIS) (Carver and White, 1994; Rizvi et al., 2016). In addition, two researcher-administered scales were completed by SZ participants only, as these are specific to the disorder. The Positive and Negative Syndrome Scale (PANSS) was used to assess the presence and severity of both positive and negative symptoms, as well as general psychopathology (Kay et al., 1987), and the Brief Negative Symptom Scale (BNSS) measured alogia, anhedonia, avolition, asociality, and blunted affect (Kirkpatrick et al., 2011).

### 2.5 Ethics

The study was reviewed and approved by a local Ethics Review Board at each of the four study sites: the Medical Ethical Review Committee of azM/Maastricht University, The Netherlands (approval number: NL69565.068.19/METC19-023); Ethics Committee of the Faculty of Medicine at Goethe University Hospital, Frankfurt, Germany (approval number: 19–200); Drug Research Ethics Committee of the Hospital del Mar, Barcelona, Spain (approval number: 8711-I); Bioethics and Ethics Committee of the School of Medicine, Aristotle University of Thessaloniki, Greece (approval number: February 28, 2020 protocol 172).

The trial was conducted in compliance with the principles of the Declaration of Helsinki (1996), the Medical Research Involving Human Subjects Act, the principles of Good Clinical Practice and in accordance with all applicable regulatory requirements including but not limited to the Research Governance Framework. The study complied with the Data Protection Act, and participant data was pseudonymised so that participants were identified only by a unique identifier.

Ethics approval was obtained for all advertisements prior to their use. Interested individuals were provided with the Participant Information Sheet and Informed Consent Form so they could make an informed decision about participation in the study. Participants were given a detailed verbal explanation of the study and written informed consent was taken prior to enrolment.

### 2.6 Sample size calculations

The sample size for RTOC was determined using power analysis. As described in the protocol manuscript (Bilderbeck et al., 2020), effect size estimates were based on Pizzagalli et al. (2008), which found a significant main effect of group (MDD, N = 23; HC, N = 25) on response bias in a probabilistic reward learning task with an effect size partial eta^2^ = 0.11. G∗Power 3.1.9.2 (Faul et al., 2009) was used to convert partial eta^2^ to Cohen's f and calculate the sample size needed to detect a statistically significant main effect of group with alpha <0.05, power = 0.95, Cohen's f = 0.3516, and number of measurements = 3 (€0.10, €0.20, €0.40). The required sample was calculated as N = 37 per group.

For their test-retest reliability analyses, Reddy et al. (2015) reported intraclass correlation coefficients (ICCs) of 0.63 and 0.59 for two GSET endpoints: (i) the difference in the percentage of hard choices for trials offering highest reward for the hard choice vs. trials offering the lowest reward for the hard choice, and (ii) the percentage of hard choices for trials offering the highest reward for the hard choice. Therefore, an ICC of 0.60 was used to calculate the sample size required to measure test-retest reliability in this study. For each group, a sample of N = 15 per group was found to be required to detect an ICC of 0.60 between the two measurement points, with p < .05 (one-tail) and power = 0.80. We targeted recruitment of N = 16 per group for retest; see also Bilderbeck et al. (2020), which describes the study protocol in more detail.

### 2.7 Statistical analysis

Statistical analysis was guided by a Statistical Analysis Plan (available here: https://osf.io/9kgpx) that was finalised prior to database lock (see Supplementary Material for deviations). The analysis was run in SPSS (version 27) and SAS 9.4. Figures were produced in Python.

The percentage of hard choices at each reward level was compared between the groups (SZ vs. SZ-HC, MDD vs. MDD-HC, SZ vs. MDD) using ANCOVAs, with group as a between-subjects factor and age, sex, and site as covariates. The effect of reward within each group was tested using Wilcoxon signed rank tests. For the within-group comparisons, non-parametric testing was chosen as the differences between the percentage of hard choices at different rewards widely deviated from normality. For both types of tests, significance values were Bonferroni corrected for three (group or reward-level) comparisons.

Twenty-six participants always chose hard trials irrespective of reward level and were classified as inflexible responders. To understand if their baseline clinical symptoms were different from those of the other ‘flexible’ responders, Mann-Whitney U tests were conducted to measure differences on the questionnaire outcomes. To assess the impact of inflexible responders, sensitivity analyses were conducted excluding these participants.

Additionally, we replicated the analysis performed by Reddy et al. (2015) on SZ participants. Reddy et al. (2015) used an ANOVA to test the main effects of reward and group and a reward-by-group interaction on the percentage of hard choices in participants with schizophrenia and healthy controls, without any covariate correction. We repeated these tests on the RTOC dataset using only participants from the SZ and SZ-HC groups, including and excluding inflexible responders.

To examine the relationship between hedonic, volitional, and clinical factors with task performance, agnostic of diagnostic group, pairwise Spearman's rank partial correlations that corrected for age, sex, and site were conducted between questionnaire and GSET outcomes. Group differences between the questionnaire outcomes were also assessed using ANCOVAs with group as a between-subjects factor and age, sex, and site as covariates. As these analyses were exploratory, they were not corrected for multiple comparisons.

As the SHAPS total score (binarised and non-binarised) negatively correlated with percentage hard choices at high reward (see Section 3.4), we investigated this relationship further by splitting participants into either low (SHAPS binarised ≤2) or high anhedonia (SHAPS binarised >2) groups, using the threshold proposed by Snaith et al. (1995) to discriminate ‘normal’ and ‘abnormal’ hedonic tone. We then tested for group differences and effects of reward within each anhedonic group using ANCOVAs and Wilcoxon signed rank tests as described above, with and without inflexible responders.

To evaluate test-retest reliability, intraclass correlations (ICCs) were calculated for the percentage of hard choices at high reward and for the difference between the percentage of hard choices at high and low reward (replicating the approach of Reddy et al. (2015)). These were computed across all participants and, separately, within each group. The ICCs were calculated using a two-way mixed-effects ANOVA and absolute agreement, then tested for statistical significance using F-tests. Practice effects between baseline and retest were evaluated using Cohen's d values and tested for statistical significance using paired t-tests. Additionally, to test if there were site effects, ANCOVAs were conducted on each GSET outcome measure with site as a between-subjects factor and age, sex, and group as covariates.

## 3 Results

### 3.1 Participants

Participant characteristics at baseline are summarised in Table 1 and at retest in Table 2. Group differences on clinical scales are reported in Supplementary Table 1. SZ and MDD participants both reported higher symptoms of depression (QIDS) than matched controls (p<.001), with MDD participants having higher symptoms than SZ (p=.043). SZ and MDD participants both also reported higher symptoms of anhedonia (SHAPS) compared to matched controls when using binarised (SZ p=.012, MDD p<.001) or non-binarised scores (SZ p=.006, MDD p<.001). The SHAPS scores of MDD participants were higher than those of SZ when using the binarised score (p=.035) and this difference was trend-level when using the non-binarised score (p=.076). MDD participants reported lower BAS drive, fun-seeking, and reward responsiveness than matched controls (all p<.001) and SZ (all p=.009), whereas there were not any statistically significant differences on these measures between SZ participants and their matched controls. SZ and MDD participants both reported higher punishment sensitivity (BIS) than matched controls (SZ p=.002, MDD p<.001), but there was not a statistically significant difference between the two clinical groups (p=.283).

**Table 1:** Participant characteristics at baseline assessment. Means (standard deviations) reported for continuous variables. Group comparisons reported in Supplementary Table 1.

	SZ	SZ-HC	MDD	MDD-HC	HC
n	37	34	40	41	59
Age	40.92 (7.62)	40.47 (9.14)	34.15 (9.78)	34.15 (9.74)	36.37 (10.30)
Sex – females, n (%)	15 (41 %)	13 (38 %)	30 (75 %)	28 (68 %)	33 (56 %)
Years of education	14.89 (3.61)	18.76 (6.17)	17.58 (3.61)	18.37 (4.85)	18.36 (4.97)
Site					
Greece	11	6	9	13	15
Netherlands	9	9	11	9	15
Germany	9	8	10	11	15
Spain	8	11	10	8	14
Medication					
Drugs for psychosis	27	0	5	0	0
Anticholinergic	3	0	0	0	0
Lithium	0	0	1	0	0
Drugs for depression	10	0	26	0	0
Drugs for epilepsy	3	0	0	0	0
Sedative	2	0	2	0	0
Questionnaires and clinical scales					
QIDS total	6.86 (4.74)	1.12 (1.09)	10.58 (6.53)	1.66 (1.30)	1.34 (1.24)
SHAPS total (binarised)	2.32 (2.93)	0.94 (1.69)	4.95 (4.16)	0.49 (1.10)	0.63 (1.36)
SHAPS total (non-binarised)	26.05 (6.36)	21.68 (6.38)	30.78 (8.38)	19.90 (4.97)	20.68 (5.52)
BAS drive	11.27 (2.14)	11.65 (2.84)	9.18 (2.92)	11.88 (2.09)	11.56 (2.49)
BAS fun seeking	11.81 (2.08)	11.59 (1.96)	10.05 (2.09)	11.98 (2.43)	11.78 (2.27)
BAS reward responsiveness	16.24 (2.35)	16.24 (2.16)	14.35 (2.29)	16.90 (1.95)	16.69 (1.98)
BIS total	21.54 (3.15)	18.65 (3.94)	23.22 (3.80)	19.02 (4.06)	19.25 (3.88)
BNSS total	21.76 (13.95)				
PANSS positive	14.14 (4.58)				
PANSS negative	17.35 (7.57)				
PANSS composite	−3.22 (6.54)				
PANSS general	32.86 (9.11)				

QIDS = Quick Inventory of Depressive Symptomatology, SHAPS = Snaith-Hamilton Pleasure Scale, BAS = Behavioural Approach System scale, BIS = Behavioural Inhibition System scale, BNSS = Brief Negative Symptom Scale, PANSS = Positive And Negative Symptom Scale.

**Table 2:** Participant characteristics at retest assessment. Means (standard deviations) reported for continuous variables.

	SZ	SZ-HC	MDD	MDD-HC	HC
n	11	10	14	14	17
Age	41.09 (7.70)	38.90 (9.07)	31.93 (9.30)	37.36 (10.48)	38.53 (10.20)
Sex – females, n (%)	4 (36 %)	3 (30 %)	10 (71 %)	9 (64 %)	10 (59 %)
Years of education	15.18 (4.12)	17.80 (4.21)	17.50 (3.78)	18.00 (3.21)	18.24 (3.53)
Site					
Greece	5	2	4	5	6
Netherlands	2	2	5	4	4
Germany	1	3	2	2	3
Spain	3	3	3	3	4

### 3.2 Percentage of hard choices

#### 3.2.1 Primary analysis

**Fig. 2:**
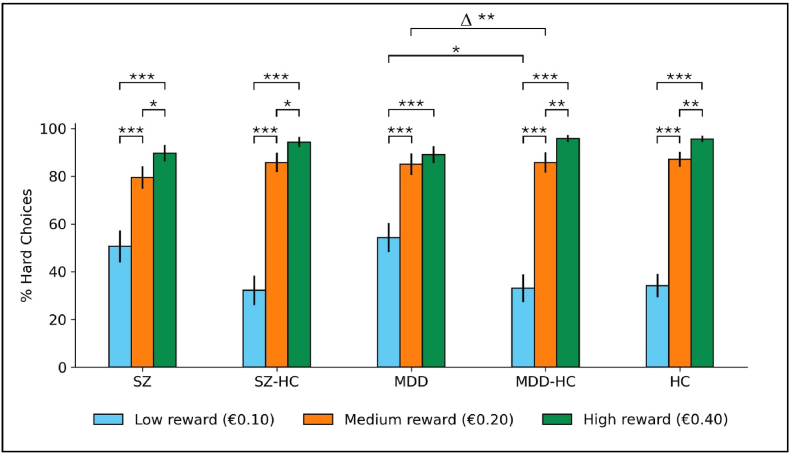
Percentage of hard choices (including inflexible responders). Δ = difference between the percentage of hard trial choices at high and low reward. ∗p < .05, ∗∗p < .01, ∗∗∗p < .001; Bonferroni corrected for 3 tests. Error bars represent ±1 standard error of the mean.

The percentage of hard choices by group and reward is shown in Fig. 2 (see Table 3 for statistically significant results, Supplementary Table 2 for all results). MDD participants selected more hard choices when offered low reward than MDD-HC (F(1,74)=7.51,p=.024, Bonf.), and showed less change in their percentage of hard choices between high and low reward (F(1,74)=12.45,p=.003, Bonf.). SZ-HC also exhibited a numerically greater difference between the percentage of hard choices at high vs. low reward when compared with SZ, but this difference was not statistically significant after Bonferroni correction (F(1,70)=1.77,p=.060, Bonf.). Within each group, except MDD, statistically significant increases in the percentage of hard choices with increasing reward were observed (Supplementary Table 3). For MDD, the difference in the percentage of hard choices between medium and high reward was not statistically significant (S=27.5,p=.153, Bonf.). The mean success rate across participants was 97.96% (98.37% in MDD, 95.75% in SZ, 99.06% in HC), indicating that the task was not too difficult.

**Table 3:** Statistically significant differences (p < .05) in percentage of hard choices between diagnostic groups and between low (SHAPS≤2) and high anhedonia (SHAPS>2) groups. All between-group comparisons reported in Supplementary Tables 2, 5, and 6, and within-group comparisons reported in Supplementary Table 3.

Reward	Group A	Group B	F (df1,df2)	p
Including inflexible responders				
Low	MDD	MDD-HC	F (1,74) = 7.51	.023^a^
High	SHAPS≤2	SHAPS>2	F (1,129) = 5.64	.019
High - Low	MDD	MDD-HC	F (1,74) = 12.45	.002^a^
	SHAPS≤2	SHAPS>2	F (1,129) = 4.13	.044
Excluding inflexible responders				
High	MDD	MDD-HC	F (1,61) = 7.13	.029^a^
	SHAPS≤2	SHAPS>2	F (1,103) = 7.29	.008
High – Low	SHAPS≤2	SHAPS>2	F (1,103) = 3.96	.049

a Bonferroni corrected for 3 tests.

#### 3.2.2 Sensitivity analysis excluding inflexible responders

**Fig. 3:**
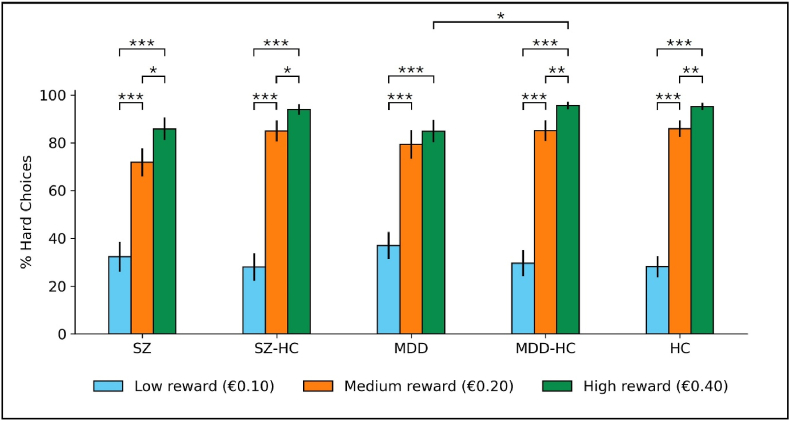
Percentage of hard choices (excluding inflexible responders). ∗p < .05, ∗∗p < .01, ∗∗∗p < .001; Bonferroni corrected for 3 tests. Error bars represent ±1 standard error of the mean.

Twenty-six participants (5 HC, 10 SZ, 11 MDD) always chose hard trials irrespective of reward level and were classified as inflexible responders. Inflexible responders reported higher QIDS total (Cohen's d=−0.36,p=.036) and BIS scores (Cohen's d=−0.49,p=0.03) than flexible responders (Supplementary Table 4, Supplementary Fig. 1). Percentages of hard choices after the inflexible responders were excluded are shown in Fig. 3. After inflexible responders were excluded, MDD participants exhibited a statistically significantly lower percentage of hard choices at high reward than MDD-HC (F(1,61)=7.13,p=.029, Bonf.; see Table 3 for statistically significant results, Supplementary Table 5 for all results). The difference in the percentage of hard choices at low reward between MDD and matched controls observed in the primary analysis was not statistically significant after inflexible responders were removed (F(1,61)=1.58,p=.642, Bonf.), suggesting that inflexible responders were driving this difference. The change in percentage of hard choices between high and low reward trials was also no longer significantly different between MDD and MDD-HC after Bonferroni correction (F(1,61)=5.73,p=.059, Bonf.). Consistent with the primary analysis, there were statistically significant increases in the percentage of hard choices with reward within each group, except between medium and high reward in MDD.

#### 3.2.3 Replication

We repeated the analysis performed by Reddy et al. (2015) on the RTOC dataset using only participants from the SZ and SZ-HC groups. Reddy et al. (2015) reported a statistically significant main effect of reward on percentage of hard choices (F(2,258)=171.9,p<.001) and a significant group-by-reward interaction (F(2,258)=3.7,p=.030), but did not find a significant main effect of group (F(1,129)=3.1,p=.080). In the RTOC dataset, when inflexible responders were included, we similarly observed a significant main effect of reward (F(2,138)=90.72,p<.001), a significant group-by-reward interaction (F(2,138)=5.994,p=.003) and no significant main effect of group (F(1,69)=.235,p=.630). When inflexible responders were excluded, there was a significant main effect of reward (F(2,114)=108.21,p<.001), but not a significant group-by-reward interaction (F(2,114)=2.18,p=.118) nor a significant main effect of group (F(1,57)=1.30,p=.258). In Reddy et al. (2015), 10% of the healthy controls (out of 40) and 8% of the SZ participants (out of 94) were reported as hard inflexible responders (3% HC were easy inflexible responders) and it was stated that the results remained largely unchanged after their exclusion.

### 3.3 High vs. low anhedonia

**Fig. 4:**
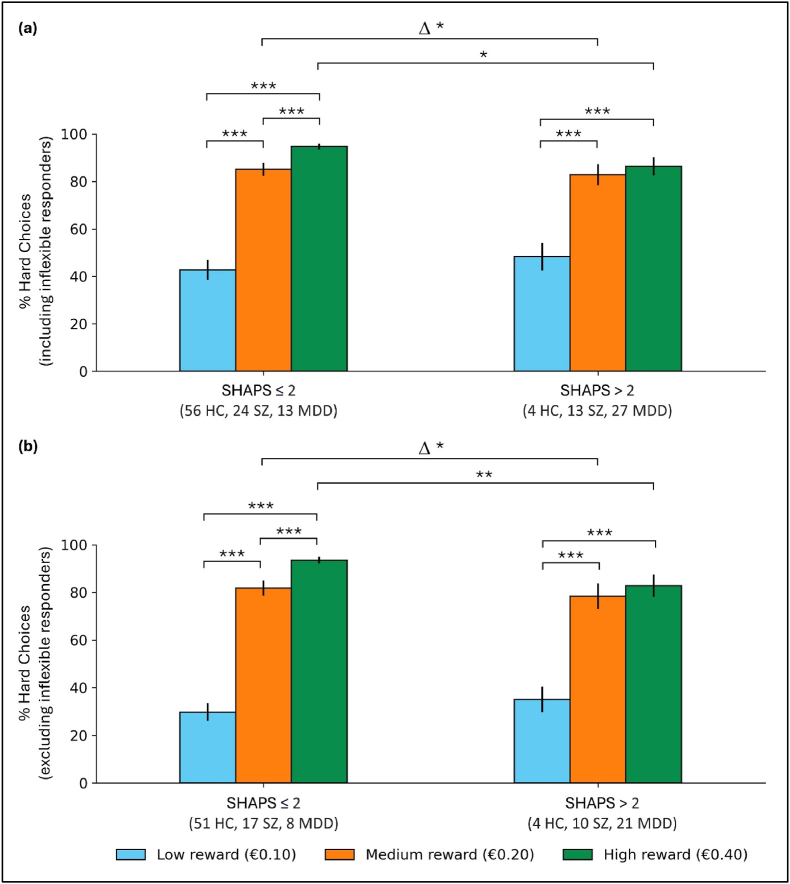
Percentage of hard choices split into low anhedonia (SHAPS ≤2) and high anhedonia (SHAPS >2) groups (a) including and (b) excluding inflexible responders. Δ = difference between the percentage of hard trial choices at high and low reward. ∗p < .05, ∗∗p < .01, ∗∗∗p < .001 (low vs. high anhedonia comparisons not corrected for multiple comparisons as a single test is performed per outcome; low vs. medium, low vs. high, medium vs. high reward comparisons Bonferroni corrected for 3 tests). Error bars represent ±1 standard error of the mean.

Ninety-three participants (56 HC, 24 SZ, 13 MDD) reported low anhedonia (SHAPS≤2) compared to 44 (4 HC, 13 SZ, 27 MDD) with high anhedonia (SHAPS>2). Fig. 4 shows the percentages of hard choices by anhedonic group (see Table 3 for statistically significant results, Supplementary Table 6 for all results). Irrespective of whether inflexible responders were included or not, the percentage of hard choices at high reward was higher for the low anhedonia group than the high anhedonia group (incl. inflex.: F(1,129)=5.61,p=.019; excl. inflex.: F(1,103)=7.29,p=.008), and the change in the percentage of hard choices at high vs. low reward was also higher for the low anhedonia group compared to the high anhedonia group (incl. inflex.: F(1,129)=4.13,p=.044; excl. inflex.: F(1,103)=3.96,p=.049). There were not any statistically significant differences between the two groups in their percentage of hard choices at low or medium reward. Increases in the percentage of hard choices with increasing reward were observed (Supplementary Table 3) except between medium and high reward in the high anhedonia group (S=29.0,p=.407, Bonf.).

### 3.4 Correlational analysis

Statistically significant correlations between questionnaire and GSET outcomes are reported in Table 4 and shown in Supplementary Fig. 2 (incl. inflex.) and Supplementary Fig. 3 (excl. inflex.), with and without covariate correction. All correlations are reported in Supplementary Table 7 (incl. inflex) and Supplementary Table 8 (excl. inflex.). The SHAPS total score negatively correlated with the percentage of hard choices at high reward, regardless of whether it was binarised $(r_{s}\left(129\right)=-0.21,p=.015$) or non-binarised ($r_{s}\left(129\right)=-0.29,p=.001)$, even after inflexible responders were removed (binarised $r_{s}\left(103\right)=-0.25,p=.009$, non-binarised $r_{s}\left(103\right)=-0.35,p<.001$). The non-binarised SHAPS total score also negatively correlated with the percentage of hard choices at medium reward ($r_{s}\left(129\right)=-0.17,p=.047)$, even after inflexible responders were excluded $\left(r_{s}\left(103\right)=-0.22,p=.023\right)$. The QIDS total score was positively correlated with the percentage of hard choices at low reward ($r_{s}\left(129\right)=0.22,p=.012$) and negatively correlated with the difference in the percentage of hard choices between high and low reward ($r_{s}\left(129\right)=-0.25,p=.004$), but these correlations were not statistically significant after inflexible responders were excluded. There was a positive correlation between BAS reward responsiveness and the percentage of hard choices at high reward after inflexible responders were excluded ($r_{s}\left(103\right)=0.22,p=.022$).

**Table 4:** Statistically significant correlations (p < .05) between questionnaires and percentage of hard choices. All correlations reported in Supplementary Tables 7 and 8

Questionnaire	Reward	r_S_ (df)	p
Including inflexible responders			
QIDS total	Low	r_S_ (129) = 0.22	0.012
	High - Low	r_S_ (129) = -0.25	0.004
SHAPS total (binarised)	High	r_S_ (129) = -0.21	0.015
SHAPS total (non-binarised)	Medium	r_S_ (129) = -0.17	0.047
	High	r_S_ (129) = -0.29	0.001
Excluding inflexible responders			
SHAPS total (binarised)	High	r_S_ (103) = -0.25	0.009
SHAPS total (non-binarised)	Medium	r_S_ (103) = -0.22	0.023
	High	r_S_ (103) = -0.35	<0.001
BAS reward responsiveness	High	r_S_ (103) = 0.22	0.022

r_S_ = Spearman's partial correlation coefficient (corrected for site, age, and sex).

### 3.5 Reliability

The intraclass correlations (ICC) and tests for practice effects, when the task is performed for the second time, are reported in Table 5. The ICCs for the percentage of hard choices at high reward and the change in the percentage of hard choices between high and low reward were statistically significant across all participants and within groups. For both outcomes, the ICCs were 0.71, showing moderate-to-high agreement between the two assessment visits, when computed across all participants. Within groups, the ICC for the percentage of hard choices at high reward was 0.59 for HC, 0.57 for SZ, and 0.85 for MDD. For the difference measure, the ICC was 0.79 for HC, 0.68 for SZ, and 0.58 for MDD. There were seven inflexible responders at retest (16.7 %), six of whom had been inflexible at baseline (Supplementary Fig. 4).

**Table 5:** Test-retest reliability and practice effects on percentage of hard choices between baseline and retest assessments.

Reward	Group	n	Test-retest reliability		Practice effects^a^	
			ICC [95 % CI]	F-test	Cohen's d [95 % CI]	*t*-test
High	All	42	0.71 [0.51, 0.83]	F (41, 41) = 5.72, p < .001	−0.08 [-0.38, 0.22]	t (41) = -0.52, p = .608
High - Low			0.71 [0.45, 0.84]	F (41, 41) = 7.03, p < .001	0.53 [0.20, 0.85]	t (41) = 3.42, p = .001
High	HC	17	0.59 [0.16, 0.83]	F (16, 16) = 3.73, p = .006	0.02 [-0.45, 0.50]	t (16) = 0.09, p = .926
High – Low			0.79 [0.48, 0.92]	F (16, 16) = 10.59, p < .001	0.55 [0.03, 1.05]	t (16) = 2.26, p = .038
High	SZ	11	0.57 [0.04, 0.86]	F (10, 10) = 3.88, p = .022	−0.44 [-1.05, 0.19]	t (10) = -1.45, p = .178
High – Low			0.68 [0.17, 0.90]	F (10, 10) = 5.00, p = .009	0.23 [-0.37, 0.83]	t (10) = 0.77, p = .458
High	MDD	14	0.85 [0.61, 0.95]	F (13, 13) = 12.22, p < .001	0.19 [-0.34, 0.72]	t (13) = 0.71, p = .488
High – Low			0.58 [0.04, 0.85]	F (13, 13) = 5.46, p = .002	0.82 [0.20, 1.42]	t (13) = 3.07, p = .009

ICC = intraclass correlation coefficient, CI = confidence interval.

a Calculated retest-baseline.

No statistically significant practice effects when performing the task the second time were observed for the percentage of hard choices at high reward. However, there were significant increases from baseline in the difference measure at retest across all participants (t(41)=3.42,p=.001), and within the HC (t(16)=2.26,p=.038) and MDD groups (t(13)=3.07,p=.009).

The effect of site (Germany, Greece, Netherlands, and Spain) on each GSET outcome was tested to evaluate if there were statistically significant differences in the outcomes that could be attributed to minor inconsistencies between how the assessments were administered (all sites completed the same training and used the same equipment) or due to language or other cultural differences. The main effect of site for each GSET outcome is reported in Supplementary Table 9. The only outcome that showed a significant site effect was the percentage of hard choices at medium reward (F(3,128)=4.39,p=.006). In ascending order, the means ± standard deviations for this outcome by site were: Netherlands = 77.3 ± 31.47, Greece = 77.46 ± 32.59, Germany = 89.22 ± 20.19, Spain = 94.62 ± 16.

## 4 Discussion

The GSET measures the willingness of participants to exert physical effort for variable amounts of monetary reward. The purpose of this task in the RTOC study was to investigate the extent to which it provides clinically meaningful behavioural biomarkers of reward processing dysfunction that differentiate SZ and MDD participants from controls, correlate with clinical scales, exhibit satisfactory test-retest reliability, and are not significantly susceptible to practice effects and site differences.

Based on previous reports (Cléry-Melin et al., 2011; Hartmann et al., 2015; Reddy et al., 2015), we hypothesised that the SZ and MDD participants would have lower percentages of hard choices than controls, especially for high reward, and less change in their percentage of hard choices between high and low reward compared to controls. While we did not see statistically significant differences between SZ participants and their matched controls, we observed that, at high reward, MDD participants were less willing to choose hard trials than controls after inflexible responders, participants who always chose hard trials irrespective of the amount of reward offered, were excluded. MDD participants also showed less change in their percentage of hard choices between high and low reward compared to controls, but this was not statistically significant after Bonferroni correction (p=.059). While, in terms of the statistical significance of the tests reported by Reddy et al. (2015), we successfully replicated their results in our SZ and SZ-HC samples, post-hoc tests did not show statistically significant case-control differences in the percentage of hard choices at any reward level. These tests are not reported in Reddy et al. (2015), but visual inspection of Figure 1 in their paper suggests it is likely they observed significantly higher percentages of hard choices at medium and high reward for controls than SZ participants, and a greater change in the percentage of hard choices from low to high reward for controls, neither of which we observed.

Our findings in the MDD group align with previous reports of reduced willingness to expend physical effort in this clinical group (Cléry-Melin et al., 2011; Treadway et al., 2012; Yang et al., 2014; Zou et al., 2020), although we note a number of studies have failed to detect case-control differences (Cathomas et al., 2021; Yang et al., 2021). The lack of statistically significant case-control differences for the SZ group in their percentage of hard choices are more at odds with the established literature (Barch et al., 2014; Gold et al., 2013; Isıklı et al., 2024; Moran et al., 2023; Reddy et al., 2015; Whitton et al., 2020) but given the pattern of our data – where percentage of hard choices was, numerically, on average lower for the SZ group than their controls at both the medium and high reward levels – it is possible that the study was underpowered to detect a significant effect, especially after the removal of inflexible responders.

The presence of inflexible responders appeared to have other effects on the behavioural observations. When inflexible responders were included in the analysis, MDD participants chose hard trials more frequently than matched controls when offered low reward and, correspondingly, showed less change in their percentage of hard choices between high and low reward. SZ participants exhibited similar trends, though these were not statistically significant (after Bonferonni correction). The greater willingness to exert effort at low reward by patients compared to controls was not expected but can be explained by the presence of inflexible responders, as this pattern was not observed once they were removed.

Few prior studies have presented data on inflexible responding. One exception, however, is the work of Reddy et al. (2015), who report rates of 10 % inflexible responding among HCs, and 8 % among SZ participants. We observed a similar frequency of inflexible responding among HCs (8.5 %) but an elevated rate among SZ (27 %) and MDD (27.5 %). The underlying reasons why participants exhibited inflexible behaviour requires further investigation, although here we observed that these individuals scored higher on measures associated with depressive symptoms (QIDS) and punishment avoidance (BIS). As such, inflexible responder status may be a marker of motivation that could help stratify heterogenous clinical groups into subgroups with distinct reward and/or effort-based decision-making impairments. Higher depressive symptoms may be indicative of reduced reward sensitivity, suggesting that inflexible responders may be less perceptive to the differences in reward amounts and hence less willing to change behaviour for the given amounts. Sensitivity to punishment is determined from endorsement of statements such as “I worry about making mistakes” and “criticism or scolding hurts me quite a bit”. Here, greater sensitivity to punishment, perhaps as part of a depressive phenotype, may be associated with persistently choosing hard trials as a way of compensating for the risk of underperforming in the task by always making the more effortful choice, even when not associated with greater reward. The modelling approaches of Saperia et al. (2023) (working with SZ, MDD and HC participants) and Whitton et al. (2024) (patients with psychosis and HCs) have suggested that performance of a subgroup of individuals is characterised by low usage of trial-wise information, such as information on reward value and required effort, leading to effort misallocation including allocation of higher effort to low reward trials. Higher levels of cognitive impairment was also a feature of this group. It is therefore possible that inflexible responding in the GSET was, at least in part, explained by individual differences in cognitive capacity, with working memory impairments contributing to highly simplified decisions (“always choose hard”) among a subset of participants. Future research should combine data on cognitive function and GSET performance to assess this possibility.

There are other, not mutually exclusive, accounts of the observed hard inflexible behaviour in the task. It is possible that the hard task was insufficiently effortful, due for example to ineffectual calibration. The high success rates that were observed might be indicative of this, although high rates of success are a desirable feature for a task which aims to present effortful but achievable targets. Insufficiently effortful hard tasks may have been better shown by grip forces substantially exceeding the target. Unfortunately, the current task lacked these recordings, as the trial progressed immediately after the participant reached the target effort (90 % of their calibrated maximum). Sites were centrally trained on task administration and no sites reported issues with calibration when collecting data. When queried about inflexible responding, sites reported back that participants had understood the task and were making their preferred choices. Nevertheless, some of the differences between the data observed here and that of, for example, Reddy et al. (2015) may be due to the differences between a single-site research study and an international multi-site initiative like the one conducted here. If there were indeed issues with site implementation, they appeared to be largely applied to all sites, as the observed inter-site differences were modest.

In terms of using the task to measure the efficacy of potential treatments for anhedonia, tasks will have diminished utility in participants with inflexible behaviour, unless the behaviour itself is shown to be a clinically meaningful symptom. We call for improved reporting of inflexible responder data to better understand this phenomenon (for studies which do report relevant data, albeit with different effort-based decision-making tasks, see also McCarthy et al. (2016) and Culbreth et al. (2024)). Semi-structured debriefing interviews or questionnaires could help assess comprehension before and after task administration (as also suggested by Whitton et al. (2024)) and could additionally be used to explore participant accounts of inflexible responding choices. Optimisations to reduce rates of inflexible behaviour may include increasing task difficulty or widening the range of rewards in terms of actual and expected values. The current task was calibrated on both the dominant and non-dominant hand – as was the case in Reddy et al. (2015) – whilst the task itself required gripping only with the dominant hand. A task which is only calibrated on the hand used in the task should represent a closer personalisation of required effort and may address ceiling effects in the behavioural data. A process could also be defined for the exclusion of calibration trials that are lower than expected grip strength values. We also suggest a modification to the task where participants are required to sustain the target grip force consistently over a short period for time (e.g., 500–1000ms) instead of simply reaching the target. This would increase the effortfulness of the task and provide richer data on participant grip strength capacity. Alternatively, the hard task could require the participant to use the non-dominant hand; similar differentiation of the easy and hard tasks is a typical feature of other effort-based decision-making tasks such as the EEfRT (Treadway et al., 2009). However, the task must be still achievable – and effects of fatigue must also be considered – so further careful testing and optimisation are clearly needed.

Across all participants, the percentage of hard choices at high reward negatively correlated with symptoms of anhedonia, and follow-up tests clearly showed expected trends where participants with high anhedonia had less willingness to exert effort at high reward compared to those with low anhedonia, and showed less change in their willingness to exert effort between low and high reward trials, regardless of whether inflexible responders were included in the analysis or not. This suggests that SZ participants with higher levels of anhedonia than reported in our sample (SHAPS M = 2.32, SD = 2.93) may be more likely to show differences from controls on the GSET. Despite having differences on all the questionnaire outcomes except the non-binarised SHAPS and BIS total, the SZ and MDD groups did not show statistically significant differences on any of the GSET outcomes, suggesting the two clinical groups show a similar behavioural phenotype in this task. It has been proposed that even though the two clinical groups may perform similarly on effort-based decision-making tasks, the mechanisms underlying the similarity in their task behaviour might be different, with the performance of SZ participants being driven more by cognitive deficits compared to reduced reward responsivity in MDD participants (Costello et al., 2024; Culbreth et al., 2018).

Across the 42 participants that performed the task at baseline and retest, we observed moderate to high test-retest reliability (ICCs = 0.71). Within groups, ICCs ranged from 0.57 to 0.85, with the highest value being in the MDD group for the percentage of hard choices at high reward. These results are broadly consistent with Reddy et al. (2015) where, within the SZ group, ICCs were 0.59 for the percentage of hard choices at high reward and 0.63 for the difference measure. The percentage of hard choices at high reward was not influenced by practice effects (performing the task a second time), however there were statistically significant increases in the difference measure when computed across all participants and when computed only within the MDD and HC groups. These findings are consistent with Reddy et al. (2015) who also observed (in an SZ sample) practice effects at retest on the difference measure, but not on the percentage of hard choices at high reward.

As this was a multi-site study across four European countries (single site per country), we investigated if there were site differences in the GSET outcomes to demonstrate task feasibility in large, international, trials. The only GSET outcome that was influenced by site was the percentage of hard choices at medium reward, with participants in Germany and Spain having higher percentage hard choices than those in the Netherlands and Greece. Since a site effect was only observed for one outcome which was not considered a primary outcome, in contrast to the percentage of hard choices at high reward or the difference measure, we conclude that it is feasible to administer the task consistently across multiple sites and countries.

A number of limitations should be noted. We aimed to replicate the analysis by Reddy et al. (2015), but sufficient statistics were not reported to conduct a power analysis on the basis of the reported data. Therefore, for effect size estimates, we used values reported by Pizzagalli et al. (2008), who used a probabilistic reward task (PRT) to assess hedonic capacity in MDD vs healthy controls. Effect sizes associated with the PRT may differ from the GSET, such that the current study may have been underpowered, particularly following the exclusion of inflexible responders from analysis. Our test-retest sample size was based on a power analysis conducted on the data of Reddy et al. (2015) and therefore this analysis should be adequately powered. However, we note the wide 95% confidence intervals for the ICC values, in particular for the within-group data (Table 5), reflecting imprecision in the ICC values which may be related to the modest sample sizes. Our study excluded MDD participants with a current episode lasting more than 6 months, to reduce clinical heterogeneity in this group. It would be of interest to examine and validate task performance in those with more chronic illness profiles, including treatment resistant depression.

## 5 Conclusions

In terms of case-control differences, the main observation from this study is that MDD patients chose fewer high-reward hard trials than controls, however only after inflexible responders were excluded. Contrary to our expectations, SZ patients did not show less willingness to exert effort than controls at any reward level or less change in their willingness to exert effort between low and high reward. However, we observed a negative correlation between willingness to exert effort at high reward and symptoms of anhedonia across all participants, and follow-up tests showed that participants with high anhedonia had a lower percentage of high-reward hard trials, and less change in their percentage of hard choices between low and high reward, than those with low anhedonia. Our results suggest that the GSET provides promising objective behavioural biomarkers related to the reward processing dysfunction, particularly anhedonia. However, the unexpectedly high proportion of inflexible responders in the patient groups may represent an unwanted ceiling effect and further investigation is needed to understand this behaviour pattern, its implications for the task's design and interpretation, and whether it should be used for participant stratification. The GSET outcomes demonstrated moderate-to-high test-retest reliability, and minimal site effects confirmed the task's operational feasibility in multi-site studies.

## Contributors

GRD and SP conceived of the research study and provided key input for the devising the study protocol, the writing of which was coordinated by AB. DH, SDV, DU, DP, BM, JT, AS, TA, OG, MMP, AR, HS, AL, GP, VP, ME, KVA, and BS contributed to the protocol design and/or data collection. AM, AB, and DH prepared the statistical analysis plan that guided the analysis. AM, AF, and SNM conducted the analysis. AM and SNM prepared the first draft of the manuscript. All authors contributed to the article and approved the submitted version.

All other authors do not report conflicts of interest.

## Role of funding source

RTOC was funded by a non-competitive consortium of the following pharmaceutical companies: 10.13039/100008349Boehringer Ingelheim International GmbH, H. Lundbeck, Janssen Pharmaceutica, BlackThorn Therapeutics, and F. Hoffmann-La Roche Ltd. The funders were involved in the study design, interpretation of data, writing of the report, and the decision to submit the paper for publication. The funders were not involved in data collection and analysis. Boehringer Ingelheim was given the opportunity to review the manuscript for medical and scientific accuracy as well as intellectual property considerations.

## Conflict of interest

AM, AB, and GRD are employees of P1vital Products Ltd. SNM, AA and AF were employees of P1vital Ltd., which was acquired by P1vital Products Ltd., when they worked on this study. GRD is an owner and shareholder of P1vital Products Ltd. SP is a former employee of Boehringer Ingelheim. KVA is an employee of Boehringer Ingelheim. DJP is a former employee of Johnson & Johnson Innovative Medicine. WJM, a former employee of BlackThorn Therapeutics, Inc., holds an ownership interest (stock) in Neumora Therapeutics, Inc. because of the acquisition of BlackThorn Therapuetics, Inc. by Neumora Therapeutics, and is a current employee and shareholder of Johnson & Johnson, Inc; each company is developing medicines to treat neuropsychiatric conditions. DU is a former employee of Hoffman La Roche Ltd.

All other authors do not report conflicts of interest.
