# Supplementary material for: Using the grip strength effort task to measure reward processing dysfunction in schizophrenia and major depressive disorder
Source: Neurosci Appl. 2025 Jul 30;4:105524. doi: 10.1016/j.nsa.2025.105524 (PMC12362125; doi:10.1016/j.nsa.2025.105524)
Supplement: Multimedia component 1 [file mmc1.docx]

**Supplementary Materials**

**Healthy control matching**

To investigate case-control differences, for each patient group (MDD, SZ), a matched HC group was constructed that consisted of a subset of the HCs that were similar to the patient group in age and sex. The process for this was defined in a statistical analysis plan (available here: <https://osf.io/9kgpx>) that was finalised prior to database lock. For each patient group, 1000 control sets were generated that were similar to the patient group in age and gender. Then, for each patient group, the ‘optimal’ control set that was closest to it in its mean age was selected. The SZ-HC and MDD-HC sets were generated independently and were not required to be mutually exclusive. This method for constructing matched control sets was deemed stable based on the following criteria: across control set generations, the ranges of the mean age and proportion of females were small and the distributions of these two parameters were unimodal and centred around single values that were close to the corresponding patient group values. The SZ and SZ-HC groups were not statistically significantly different in age ($t(69)= 0.225, p= .823$) or sex ($\chi^{2}(1)= 0.039, p= .843$). Similarly, the MDD and MDD-HCs were not significantly different in age ($t(79)= 0.002$, $p= .999)$or sex ($\chi^{2}(1)= 0.448, p= .503$).

**Calculation of SHAPS total scores**

The standard scoring of SHAPS is calculating using binarised responses. Each of the 14 items in the SHAPS has four possible responses: ‘definitely disagree’, ‘disagree’, ‘agree’, or ‘definitely agree’. In binarised scoring, both ‘disagree’ responses are scored with one point and both ‘agree’ responses with zero points (Snaith et al., 1995). In RTOC, we additionally computed a non-binarised score (‘definitely disagree’ = 4, ‘disagree’ = 3, ‘agree’ = 2, and ‘definitely agree’ = 1) in order to capture more variability in participants’ responses.

**Deviations from the statistical analysis plan (SAP)**

The RTOC statistical analysis plan (SAP) was finalised on 15JAN2021, prior to database lock (available here: <https://osf.io/9kgpx>). There are some deviations from the planned analysis in how the analysis has been conducted for this manuscript. These deviations, and the reasons for them, are described below.

There were 26 inflexible responders that always chose hard trials irrespective of the reward level. To assess the extent to which the results changed when these participants were excluded, sensitivity analyses that excluded inflexible responders were conducted. Additionally, to understand differences between the ‘flexible’ and inflexible responders, statistical significance tests were performed on questionnaire scores and calibration grip strength. In the SAP, neither of these analyses had been specified as the relatively high number of inflexible responders had not been anticipated.

In Section 8 of the SAP, it was specified that the test-retest and practice effects analyses would be performed for each group separately. However, in addition to this, we performed this analysis across all groups as well. The reasons for this were: (i) sample sizes within groups were small (ranging from 11 to 17) and, therefore, it was decided that looking at the reliability across groups would be more robust, and (ii) looking at test-retest across all participants would be transdiagnostic.

In the SAP, the difference between the percentage of hard choices at high and low reward was the only behavioural GSET outcome proposed for the test-retest and practice effects analyses (SAP Table 8). We additionally conducted these analyses on the percentage of hard choices at high reward as well as it had been similarly examined in Reddy et al. (2015).

In Section 5.1 of the SAP, it was stated that the comparison between the SZ and MDD groups would be performed by first testing for a main effect of group in an ANCOVA that included all participants and then performing a post-hoc comparison between the two groups if the main effect was significant. In the analysis, this two-step procedure was not followed. Instead, the comparison between the SZ and MDD groups was performed by testing for a main effect of group in an ANCOVA that only included the SZ and MDD participants. The reason for this was that it was decided that the post-hoc comparison was interesting to perform, regardless of whether the main effect of group across all participants was significant or not, given the exploratory nature of the study and the significance of identifying biomarkers that can potentially distinguish the two patient groups.

The partial correlations conducted between the questionnaire scores and GSET outcomes included sex as a covariate, which was not mentioned in the SAP (SAP section 5.2). This was an oversight during the development of the SAP as the mention of this covariate was accidently missed - it had always been the intention to include sex with the other covariates.

Years of education was not used as a covariate in the analyses, even though this was specified in Sections 5.1 and 5.2 of the SAP. The reason for this was that, after the study had completed, it came to our attention that this variable had been calculated inconsistently across sites.

Group differences between the questionnaire outcomes were assessed using ANCOVAs. These group comparisons had not been considered during SAP development, but it was decided to conduct these for the manuscript to indicate where the between-group differences were statistically significant to complement descriptive statistics.

To further inspect the negative correlation between the SHAPS and percentage of hard choices, each GSET outcome was compared between high and low anhedonia groups. This analysis had not been proposed in the SAP.

**Supplementary Tables**

*Supplementary Table 1*

*Supplementary Table 1 – Differences between the groups on clinical scales.*

|  |  |  |  |  | Difference (Group A – Group B) | | |
| --- | --- | --- | --- | --- | --- | --- | --- |
| Scale | Group A | LSMean (SE) | Group B | LSMean (SE) | LSMean (SE) | F(df_1_,df_2_) | p |
| QIDS total | SZ | 6.705 (0.568) | SZ-HC | 1.291 (0.593) | 5.414 (0.827) | F(1,64)=42.88 | <.001 |
|  | MDD | 10.531 (0.690) | MDD-HC | 1.701 (0.682) | 8.830 (0.975) | F(1,74)=81.96 | <.001 |
|  | SZ | 7.317 (0.934) | MDD | 10.157 (0.893) | -2.840 (1.378) | F(1,70)=4.25 | .043 |
| SHAPS total (binarised) | SZ | 2.373 (0.394) | SZ-HC | 0.888 (0.411) | 1.486 (0.573) | F(1,64)=6.72 | .012 |
|  | MDD | 4.864 (0.464) | MDD-HC | 0.572 (0.459) | 4.292 (0.656) | F(1,74)=42.76 | <.001 |
|  | SZ | 2.693 (0.606) | MDD | 4.609 (0.579) | -1.916 (0.893) | F(1,70)=4.60 | .035 |
| SHAPS total (non-binarised) | SZ | 25.974 (1.012) | SZ-HC | 21.764 (1.057) | 4.210 (1.474) | F(1,64)=8.16 | .006 |
|  | MDD | 30.717 (1.049) | MDD-HC | 19.959 (1.036) | 10.758 (1.483) | F(1,74)=52.64 | <.001 |
|  | SZ | 26.824 (1.219) | MDD | 30.063 (1.166) | -3.240 (1.798) | F(1,70)=3.25 | .076 |
| BAS drive | SZ | 11.285 (0.409) | SZ-HC | 11.631 (0.427) | -0.346 (0.596) | F(1,64)=0.34 | .564 |
|  | MDD | 9.163 (0.410) | MDD-HC | 11.889 (0.405) | -2.726 (0.579) | F(1,74)=22.15 | <.001 |
|  | SZ | 11.102 (0.444) | MDD | 9.331 (0.424) | 1.770 (0.654) | F(1,70)=7.32 | .009 |
| BAS fun seeking | SZ | 11.821 (0.342) | SZ-HC | 11.577 (0.357) | 0.244 (0.499) | F(1,64)=0.24 | .626 |
|  | MDD | 10.090 (0.366) | MDD-HC | 11.937 (0.361) | -1.847 (0.517) | F(1,74)=12.75 | <.001 |
|  | SZ | 11.655 (0.371) | MDD | 10.194 (0.355) | 1.462 (0.548) | F(1,70)=7.12 | .009 |
| BAS reward responsive-ness | SZ | 16.279 (0.384) | SZ-HC | 16.196 (0.401) | 0.084 (0.559) | F(1,64)=0.02 | .882 |
|  | MDD | 14.377 (0.335) | MDD-HC | 16.876 (0.331) | -2.499 (0.474) | F(1,74)=27.76 | <.001 |
|  | SZ | 16.131 (0.422) | MDD | 14.454 (0.403) | 1.677 (0.622) | F(1,70)=7.26 | .009 |
| BIS total | SZ | 21.493 (0.594) | SZ-HC | 18.698 (0.620) | 2.795 (0.865) | F(1,64)=10.44 | .002 |
|  | MDD | 23.237 (0.606) | MDD-HC | 19.012 (0.598) | 4.225 (0.856) | F(1,74)=24.37 | <.001 |
|  | SZ | 21.906 (0.614) | MDD | 22.887 (0.587) | -0.981 (0.905) | F(1,70)=1.17 | .283 |

*Abbreviations:* LSMean = least squares mean, SE = standard error, df = degrees of freedom, SZ = schizophrenia, MDD = major depressive disorder, HC = healthy controls, SZ-HC = subset of HC matched to SZ, MDD-HC = subset of HC matched to MDD, QIDS = quick inventory of depressive symptomatology (16-item self-report), SHAPS = Snaith-Hamilton pleasure scale, BAS = behavioural approach system scale, BIS = behavioural inhibition system scale

*Supplementary Table 2*

*Supplementary Table 2 – Differences between the groups in percentage of hard choices (including inflexible responders). Statistically significant (p<.05) results highlighted in bold.*

|  |  | |  | | Difference (Group A – Group B) | | |
| --- | --- | --- | --- | --- | --- | --- | --- |
| Reward level | Group A | LSMean (SE) | Group B | LSMean (SE) | LSMean (SE) | F(df_1_,df_2_) | p (Bonf.) |
| Low (€0.10) | SZ | 50.266 (6.303) | SZ-HC | 32.554 (6.579) | 17.711 (9.178) | F(1,64)=3.72 | .174 |
|  | **MDD** | **54.529 (5.597)** | **MDD-HC** | **32.844 (5.528)** | **21.685 (7.911)** | **F(1,74)=7.51** | **.023** |
|  | SZ | 44.004 (6.705) | MDD | 60.408 (6.414) | -16.404 (9.891) | F(1,70)=2.75 | .305 |
| Medium (€0.20) | SZ | 79.875 (4.224) | SZ-HC | 85.300 (4.409) | -5.425 (6.151) | F(1,64)=0.78 | >.999 |
|  | MDD | 84.106 (4.225) | MDD-HC | 86.645 (4.173) | -2.540 (5.972) | F(1,74)=0.18 | >.999 |
|  | SZ | 77.338 (5.027) | MDD | 86.935 (4.809) | -9.596 (7.415) | F(1,70)=1.67 | .600 |
| High (€0.40) | SZ | 89.477 (2.902) | SZ-HC | 94.458 (3.029) | -4.981 (4.226) | F(1,64)=1.39 | .729 |
|  | MDD | 88.957 (2.685) | MDD-HC | 95.868 (2.652) | -6.912 (3.796) | F(1,74)=3.32 | .218 |
|  | SZ | 87.842 (3.878) | MDD | 90.690 (3.710) | -2.848 (5.720) | F(1,70)=0.25 | >.999 |
| High - Low | SZ | 39.211 (6.524) | SZ-HC | 61.903 (6.810) | -22.692 (9.500) | F(1,64)=5.71 | .060 |
|  | **MDD** | **34.427 (5.735)** | **MDD-HC** | **63.024 (5.664)** | **-28.597 (8.106)** | **F(1,74)=12.45** | **.002** |
|  | SZ | 43.838 (6.912) | MDD | 30.282 (6.613) | 13.556 (10.196) | F(1,70)=1.77 | .564 |

*Abbreviations:* LSMean = least squares mean, SE = standard error, df = degrees of freedom

*Supplementary Table 3*

*Supplementary Table 3 – Within-group comparisons of percentage of hard choices between each reward level.*

| Group |  | Reward A | Median  (Q1,Q3) | Reward B | Median  (Q1,Q3) | S | p (Bonf.) |
| --- | --- | --- | --- | --- | --- | --- | --- |
| SZ | | | | | | | |
|  | Including inflexible responders | Low | 50.00 (11.11,100.00) | High | 100.00 (94.44,100.00) | 174.5 | <.001 |
|  |  | Low | 50.00 (11.11,100.00) | Medium | 100.00 (61.11,100.00) | 163.0 | <.001 |
|  |  | Medium | 100.00 (61.11,100.00) | High | 100.00 (94.44,100.00) | 42.5 | .015 |
|  | Excluding inflexible responders | Low | 16.67 (5.56,55.56) | High | 100.00 (77.78,100.00) | 174.5 | <.001 |
|  |  | Low | 16.67 (5.56,55.56) | Medium | 83.33 (44.44,100.00) | 163.0 | <.001 |
|  |  | Medium | 83.33 (44.44,100.00) | High | 100.00 (77.78,100.00) | 42.5 | .015 |
| MDD | | | | | | | |
|  | Including inflexible responders | Low | 47.22 (16.67,100.00) | High | 100.00 (91.67,100.00) | 187.0 | <.001 |
|  |  | Low | 47.22 (16.67,100.00) | Medium | 100.00 (86.11,100.00) | 163.0 | <.001 |
|  |  | Medium | 100.00 (86.11,100.00) | High | 100.00 (91.67,100.00) | 27.5 | .153 |
|  | Excluding inflexible responders | Low | 33.33 (11.11,61.11) | High | 100.00 (77.78,100.00) | 187.0 | <.001 |
|  |  | Low | 33.33 (11.11,61.11) | Medium | 100.00 (66.67,100.00) | 163.0 | <.001 |
|  |  | Medium | 100.00 (66.67,100.00) | High | 100.00 (77.78,100.00) | 27.5 | .153 |
| HC | | | | | | | |
|  | Including inflexible responders | Low | 16.67 (0.00,72.22) | High | 100.00 (94.44,100.00) | 707.0 | <.001 |
|  |  | Low | 16.67 (0.00,72.22) | Medium | 100.00 (88.89,100.00) | 709.5 | <.001 |
|  |  | Medium | 100.00 (88.89,100.00) | High | 100.00 (94.44,100.00) | 88.0 | .002 |
|  | Excluding inflexible responders | Low | 11.11 (0.00,44.44) | High | 100.00 (94.44,100.00) | 707.0 | <.001 |
|  |  | Low | 11.11 (0.00,44.44) | Medium | 100.00 (77.78,100.00) | 709.5 | <.001 |
|  |  | Medium | 100.00 (77.78,100.00) | High | 100.00 (94.44,100.00) | 88.0 | .002 |
| SZ-HC | | | | | | | |
|  | Including inflexible responders | Low | 13.89 (0.00,66.67) | High | 100.00 (94.44,100.00) | 259.0 | <.001 |
|  |  | Low | 13.89 (0.00,66.67) | Medium | 100.00 (77.78,100.00) | 251.5 | <.001 |
|  |  | Medium | 100.00 (77.78,100.00) | High | 100.00 (94.44,100.00) | 46.5 | .017 |
|  | Excluding inflexible responders | Low | 11.11 (0.00,47.22) | High | 100.00 (94.44,100.00) | 259.0 | <.001 |
|  |  | Low | 11.11 (0.00,47.22) | Medium | 100.00 (72.23,100.00) | 251.5 | <.001 |
|  |  | Medium | 100.00 (72.23,100.00) | High | 100.00 (94.44,100.00) | 46.5 | .017 |
| MDD-HC | | | | | | | |
|  | Including inflexible responders | Low | 16.67 (5.56,66.67) | High | 100.00 (100.00,100.00) | 363.0 | <.001 |
|  |  | Low | 16.67 (5.56,66.67) | Medium | 100.00 (94.44,100.00) | 363.0 | <.001 |
|  |  | Medium | 100.00 (94.44,100.00) | High | 100.00 (100.00,100.00) | 40.5 | .009 |
|  | Excluding inflexible responders | Low | 11.11 (0.00,55.56) | High | 100.00 (94.44,100.00) | 363.0 | <.001 |
|  |  | Low | 11.11 (0.00,55.56) | Medium | 100.00 (66.67,100.00) | 363.0 | <.001 |
|  |  | Medium | 100.00 (66.67,100.00) | High | 100.00 (94.44,100.00) | 40.5 | .009 |
| Low anhedonia (SHAPS ≤ 2) | | | | | | | |
|  | Including inflexible responders | Low | 25.00 (5.56,88.89) | High | 100.00 (100.00,100.00) | 1374.5 | <.001 |
|  |  | Low | 25.00 (5.56,88.89) | Medium | 100.00 (75.00,100.00) | 1332.5 | <.001 |
|  |  | Medium | 100.00 (75.00,100.00) | High | 100.00 (100.00,100.00) | 242.0 | <.001 |
|  | Excluding inflexible responders | Low | 16.67 (5.56,50.00) | High | 100.00 (94.44,100.00) | 1374.5 | <.001 |
|  |  | Low | 16.67 (5.56,50.00) | Medium | 100.00 (66.67,100.00) | 1332.5 | <.001 |
|  |  | Medium | 100.00 (66.67,100.00) | High | 100.00 (94.44,100.00) | 242.0 | <.001 |
| High anhedonia (SHAPS > 2) | | | | | | | |
|  | Including inflexible responders | Low | 38.89 (13.89,94.44) | High | 100.00 (88.89,100.00) | 260.5 | <.001 |
|  |  | Low | 38.89 (13.89,94.44) | Medium | 100.00 (72.23,100.00) | 236.0 | <.001 |
|  |  | Medium | 100.00 (72.23,100.00) | High | 100.00 (88.89,100.00) | 29.0 | .407 |
|  | Excluding inflexible responders | Low | 27.78 (11.11,55.56) | High | 94.44 (83.33,100.00) | 260.5 | <.001 |
|  |  | Low | 27.78 (11.11,55.56) | Medium | 100.00 (50.00,100.00) | 236.0 | <.001 |
|  |  | Medium | 100.00 (50.00,100.00) | High | 94.44 (83.33,100.00) | 29.0 | .407 |

*Abbreviations:* Q1 = lower quartile (25%), Q3 = upper quartile (75%), S = Wilcoxon signed rank test statistic, low reward = €0.10, medium reward = €0.20, high reward = €0.40, SHAPS = Snaith-Hamilton pleasure scale.

*Supplementary Table 4*

*Supplementary Table 4 – Comparison of participant characteristics of ‘flexible’ and inflexible responders at baseline. Means (standard deviations) reported for continuous variables.*

|  | | | All | | | | SZ | | MDD | | HC | |
| --- | --- | --- | --- | --- | --- | --- | --- | --- | --- | --- | --- | --- |
|  | | | Flexible | Inflexible | Cohen’s d | MWU ^1^ | Flexible | Inflexible | Flexible | Inflexible | Flexible | Inflexible |
| n | | | 110 | 26 |  |  | 27 | 10 | 29 | 11 | 54 | 5 |
| Age | | | 35.96 (9.75) | 41.15 (8.81) |  |  | 39.11 (7.79) | 45.80 (4.54) | 32.59 (9.39) | 38.27 (10.01) | 36.20 (10.39) | 38.20 (10.03) |
| Sex | | | 63 | 15 |  |  | 11 | 4 | 22 | 8 | 30 | 3 |
| Years of education | | | 17.09 (4.45) | 17.58 (4.61) |  |  | 14.74 (3.60) | 15.30 (3.80) | 17.14 (3.70) | 18.73 (3.23) | 18.24 (4.79) | 19.60 (7.23) |
| Site | | |  |  |  |  |  |  |  |  |  |  |
|  | Greece | | 25 | 10 |  |  | 6 | 5 | 5 | 4 | 14 | 1 |
|  | Netherlands | | 28 | 7 |  |  | 6 | 3 | 8 | 3 | 14 | 1 |
|  | Germany | | 31 | 3 |  |  | 9 | 0 | 7 | 3 | 15 | 0 |
|  | Spain | | 26 | 6 |  |  | 6 | 2 | 9 | 1 | 11 | 3 |
| Medication | |  |  |  |  |  |  |  |  |  |  |  |
|  | Antipsychotic | | 23 | 9 |  |  | 18 | 9 | 5 | 0 | 0 | 0 |
|  | Anticholinergic | | 2 | 1 |  |  | 2 | 1 | 0 | 0 | 0 | 0 |
|  | Lithium | | 1 | 0 |  |  | 0 | 0 | 1 | 0 | 0 | 0 |
|  | Antidepressant | | 28 | 8 |  |  | 6 | 4 | 22 | 4 | 0 | 0 |
|  | Antiepileptic | | 3 | 0 |  |  | 3 | 0 | 0 | 0 | 0 | 0 |
|  | Sedative | | 3 | 1 |  |  | 1 | 1 | 2 | 0 | 0 | 0 |
| Questionnaire | |  |  |  |  |  |  |  |  |  |  |  |
|  | **QIDS total** | | **5.15 (5.83)** | **7.27 (5.94)** | **-0.36** | **Z=2.10, p=.036** | 6.85 (4.88) | 6.90 (4.58) | 10.62 (6.77) | 10.45 (6.15) | 1.37 (1.26) | 1.00 (1.00) |
|  | SHAPS total (binarised) | | 2.41 (3.39) | 2.15 (3.37) | 0.08 | Z=-0.50, p=.619 | 2.78 (3.19) | 1.10 (1.60) | 5.31 (4.09) | 4.00 (4.38) | 0.67 (1.41) | 0.20 (0.45) |
|  | SHAPS total (non-binarised) | | 25.20 (7.94) | 24.73 (7.90) | 0.06 | Z=-0.24, p=.810 | 27.04 (6.40) | 23.40 (5.72) | 31.76 (7.95) | 28.18 (9.31) | 20.76 (5.55) | 19.80 (5.67) |
|  | BAS drive | | 10.74 (2.59) | 10.96 (3.30) | -0.08 | Z=0.73, p=.464 | 10.85 (2.16) | 12.40 (1.71) | 9.14 (2.55) | 9.27 (3.88) | 11.54 (2.45) | 11.80 (3.19) |
|  | BAS fun seeking | | 11.28 (2.37) | 11.27 (1.99) | 0.01 | Z=-0.22, p=.826 | 11.70 (2.07) | 12.10 (2.18) | 10.07 (2.30) | 10.00 (1.48) | 11.72 (2.36) | 12.40 (0.89) |
|  | BAS reward responsiveness | | 16.00 (2.33) | 15.38 (2.62) | 0.26 | Z=-1.14, p=.254 | 16.44 (2.33) | 15.70 (2.45) | 14.41 (2.29) | 14.18 (2.40) | 16.63 (1.96) | 17.40 (2.41) |
|  | **BIS total** | | **20.67 (4.08)** | **22.62 (3.36)** | **-0.49** | **Z=2.17, p=.030** | 21.19 (3.23) | 22.50 (2.84) | 23.28 (3.82) | 23.09 (3.94) | 19.02 (3.85) | 21.80 (3.49) |
|  | BNSS total | |  |  |  |  | 20.56 (14.15) | 25.00 (13.56) |  |  |  |  |
|  | PANSS positive | |  |  |  |  | 13.70 (4.81) | 15.30 (3.89) |  |  |  |  |
|  | PANSS negative | |  |  |  |  | 16.59 (7.85) | 19.40 (6.72) |  |  |  |  |
|  | PANSS composite | |  |  |  |  | -2.89 (6.57) | -4.10 (6.74) |  |  |  |  |
|  | PANSS general | |  |  |  |  | 32.37 (9.89) | 34.20 (6.86) |  |  |  |  |

*Abbreviations:* SZ = schizophrenia, MDD = major depressive disorder, HC = healthy controls, MWU = Mann-Whitney U test, QIDS = quick inventory of depressive symptomatology (16-item self-report), SHAPS = Snaith-Hamilton pleasure scale, BAS = behavioural approach system scale, BIS = behavioural inhibition system scale, BNSS = brief negative symptom scale, PANSS = positive and negative symptom scale.

^1^ Mann-Whitney U tests were conducted to test for differences between the questionnaire scores available for all participant groups.

*Supplementary Table 5*

*Supplementary Table 5 – Differences between the groups in percentage of hard choices (excluding inflexible responders). Statistically significant (p<.05) results highlighted in bold.*

|  |  | |  | | Difference (Group A – Group B) | | |
| --- | --- | --- | --- | --- | --- | --- | --- |
| Reward level | Group A | LSMean (SE) | Group B | LSMean (SE) | LSMean (SE) | F(df_1_,df_2_) | p (Bonf.) |
| Low (€0.10) | SZ | 32.264 (6.215) | SZ-HC | 27.986 (5.705) | 4.279 (8.468) | F(1,52)=0.26 | >.999 |
|  | MDD | 38.340 (5.803) | MDD-HC | 28.614 (4.985) | 9.726 (7.745) | F(1,61)=1.58 | .642 |
|  | SZ | 28.523 (6.627) | MDD | 40.495 (6.359) | -11.972 (9.873) | F(1,49)=1.47 | .693 |
| Medium (€0.20) | SZ | 71.794 (4.923) | SZ-HC | 84.910 (4.519) | -13.116 (6.707) | F(1,52)=3.82 | .168 |
|  | MDD | 77.547 (5.103) | MDD-HC | 86.355 (4.384) | -8.808 (6.811) | F(1,61)=1.67 | .602 |
|  | SZ | 70.983 (6.261) | MDD | 80.082 (6.007) | -9.099 (9.327) | F(1,49)=0.95 | >.999 |
| High (€0.40) | SZ | 85.903 (3.650) | SZ-HC | 93.838 (3.351) | -7.935 (4.974) | F(1,52)=2.55 | .350 |
|  | **MDD** | **84.348 (3.261)** | **MDD-HC** | **95.969 (2.802)** | **-11.620 (4.352)** | **F(1,61)=7.13** | **.029** |
|  | SZ | 84.835 (5.216) | MDD | 85.766 (5.005) | -0.932 (7.771) | F(1,49)=0.01 | >.999 |
| High - Low | SZ | 53.638 (7.088) | SZ-HC | 65.852 (6.507) | -12.214 (9.657) | F(1,52)=1.60 | .635 |
|  | MDD | 46.008 (6.680) | MDD-HC | 67.354 (5.739) | -21.346 (8.916) | F(1,61)=5.73 | .059 |
|  | SZ | 56.312 (8.209) | MDD | 45.272 (7.877) | 11.040 (12.229) | F(1,49)=0.82 | >.999 |

*Abbreviations:* LSMean = least squares mean, SE = standard error, df = degrees of freedom

*Supplementary Table 6*

*Supplementary Table 6 – Differences between low anhedonia (SHAPS ≤ 2) and high anhedonia (SHAPS > 2) groups. Statistically significant (p<.05) results highlighted in bold.*

|  | Reward level | SHAPS ≤ 2 LSMean (SE) | SHAPS > 2 LSMean (SE) | Difference | | |
| --- | --- | --- | --- | --- | --- | --- |
|  |  |  |  | LSMean (SE) | F(df1,df2) | p |
| Including inflexible responders | | | | | | |
|  | Low (€0.10) | 42.229  (4.024) | 49.456  (5.859) | -7.227  (7.168) | F(1,129)=1.02 | .315 |
|  | Medium (€0.20) | 85.174  (2.736) | 82.767  (3.983) | 2.407  (4.873) | F(1,129)=0.24 | .622 |
|  | **High (€0.40)** | **94.570**  **(1.853)** | **86.732**  **(2.698)** | **7.837**  **(3.301)** | **F(1,129)=5.64** | **.019** |
|  | **High -**  **Low** | **52.340**  **(4.164)** | **37.276**  **(6.062)** | **15.064**  **(7.417)** | **F(1,129)=4.13** | **.044** |
| Excluding inflexible responders | | | | | | |
|  | Low (€0.10) | 30.022  (3.636) | 34.556  (5.376) | -4.533  (6.568) | F(1,103)=0.48 | .492 |
|  | Medium (€0.20) | 82.520  (3.154) | 76.824  (4.664) | 5.696  (5.697) | F(1,103)=1.00 | .320 |
|  | **High (€0.40)** | **93.582**  **(2.211)** | **82.799**  **(3.269)** | **10.783**  **(3.993)** | **F(1,103)=7.29** | **.008** |
|  | **High -**  **Low** | **63.560**  **(4.259)** | **48.243**  **(6.297)** | **15.316**  **(7.693)** | **F(1,103)=3.96** | **.049** |

*Abbreviations:* SHAPS = Snaith-Hamilton pleasure scale, LSMean = least squares mean, SE = standard error, df = degrees of freedom

*Supplementary Table 7*

*Supplementary Table 7 – Spearman’s rank partial correlations (corrected for site, age, and sex) between questionnaire outcomes and GSET outcomes (including inflexible responders). Statistically significant (p<.05) results highlighted in bold.*

| Questionnaire outcome | Low  reward (€0.10) | Medium reward (€0.20) | High  reward (€0.40) | High – Low reward |
| --- | --- | --- | --- | --- |
| QIDS total | **r_S_(129)=0.22** | r_S_(129)=-0.03 | r_S_(129)=-0.04 | **r_S_(129)=-0.25** |
|  | **p=.012** | p=.699 | p=.631 | **p=.004** |
| SHAPS total (binarised) | r_S_(129)=0.04 | r_S_(129)=-0.12 | **r_S_(129)=-0.21** | r_S_(129)=-0.13 |
|  | p=.641 | p=.176 | **p=.015** | p=.139 |
| SHAPS total (non-binarised) | r_S_(129)=0.04 | **r_S_(129)=-0.17** | **r_S_(129)=-0.29** | r_S_(129)=-0.14 |
|  | p=.614 | **p=.047** | **p=.001** | p=.099 |
| BAS drive | r_S_(129)=0.07 | r_S_(129)=0.09 | r_S_(129)=0.13 | r_S_(129)=-0.03 |
|  | p=.395 | p=.294 | p=.148 | p=.772 |
| BAS fun seeking | r_S_(129)=0.01 | r_S_(129)=-0.03 | r_S_(129)=0.04 | r_S_(129)=0.00 |
|  | p=.895 | p=.747 | p=.617 | p=.970 |
| BAS reward responsiveness | r_S_(129)=-0.08 | r_S_(129)=0.08 | r_S_(129)=0.15 | r_S_(129)=0.11 |
|  | p=.378 | p=.383 | p=.082 | p=.195 |
| BIS total | r_S_(129)=0.12 | r_S_(129)=0.07 | r_S_(129)=0.11 | r_S_(129)=-0.09 |
|  | p=.182 | p=.450 | p=.208 | p=.308 |

*Abbreviations:* r_S_ = Spearman’s correlation coefficient, df = degrees of freedom, QIDS = Quick Inventory of Depressive Symptomatology (16-item self-report), SHAPS = Snaith-Hamilton Pleasure Scale, BAS = Behavioural Approach System scale, BIS = Behavioural Inhibition System scale.

*Supplementary Table 8*

*Supplementary Table 8 – Spearman’s rank partial correlations (corrected for site, age, and sex) between questionnaire outcomes and GSET outcomes (excluding inflexible responders). Statistically significant (p<.05) results highlighted in bold.*

| Questionnaire outcome | Low  reward (€0.10) | Medium reward (€0.20) | High  reward (€0.40) | High – Low reward |
| --- | --- | --- | --- | --- |
| QIDS total | r_S_(103)=0.10 | r_S_(103)=-0.13 | r_S_(103)=-0.12 | r_S_(103)=-0.14 |
|  | p=.316 | p=.187 | p=.218 | p=.154 |
| SHAPS total (binarised) | r_S_(103)=0.07 | r_S_(103)=-0.15 | **r_S_(103)=-0.25** | r_S_(103)=-0.16 |
|  | p=.509 | p=.137 | **p=.009** | p=.100 |
| SHAPS total (non-binarised) | r_S_(103)=0.05 | **r_S_(103)=-0.22** | **r_S_(103)=-0.35** | r_S_(103)=-0.17 |
|  | p=.602 | **p=.023** | **p<.001** | p=.078 |
| BAS drive | r_S_(103)=0.08 | r_S_(103)=0.10 | r_S_(103)=0.14 | r_S_(103)=-0.03 |
|  | p=.419 | p=.314 | p=.142 | p=.771 |
| BAS fun seeking | r_S_(103)=0.05 | r_S_(103)=-0.01 | r_S_(103)=0.06 | r_S_(103)=-0.02 |
|  | p=.583 | p=.905 | p=.518 | p=.819 |
| BAS reward responsiveness | r_S_(103)=-0.01 | r_S_(103)=0.14 | **r_S_(103)=0.22** | r_S_(103)=0.07 |
|  | p=.901 | p=.149 | **p=.022** | p=.490 |
| BIS total | r_S_(103)=-0.02 | r_S_(103)=-0.00 | r_S_(103)=0.06 | r_S_(103)=0.03 |
|  | p=.801 | p=.981 | p=.553 | p=.752 |

*Abbreviations: :* r_S_ = Spearman’s correlation coefficient, df = degrees of freedom, QIDS = Quick Inventory of Depressive Symptomatology (16-item self-report), SHAPS = Snaith-Hamilton Pleasure Scale, BAS = Behavioural Approach System scale, BIS = Behavioural Inhibition System scale.

*Supplementary Table 9*

*Supplementary Table 9 – Comparison of percentage of hard trial choices by site. Means (standard deviations) are reported for each site. Statistically significant (p<.05) results highlighted in bold.*

| Reward | F(df_1_,df_2_) | p | ηp2 | DE | ES | NL | GR |
| --- | --- | --- | --- | --- | --- | --- | --- |
| Low (€0.10) | F(128,3)=0.43 | .730 | 0.01 | 44.12 (39.59) | 38.02 (38.90) | 46.83 (38.31) | 48.73 (41.75) |
| **Medium (€0.20)** | **F(128,3)=4.39** | **.006** | **0.09** | 89.22 (20.19) | 94.62 (16.00) | 77.30 (31.47) | 77.46 (32.59) |
| High (€0.40) | F(128,3)=1.44 | .234 | 0.03 | 91.50 (20.80) | 96.70 (10.83) | 87.62 (22.59) | 92.70 (14.25) |
| High – Low | F(128,3)=1.29 | .280 | 0.03 | 47.39 (41.89) | 58.68 (39.46) | 40.79 (41.46) | 43.97 (41.49) |

*Abbreviations:* ηp2 = partial eta squared, DE = Germany, ES = Spain, NL = The Netherlands, GR = Greece.

**Supplementary Figures**

*Supplementary Figure 1*


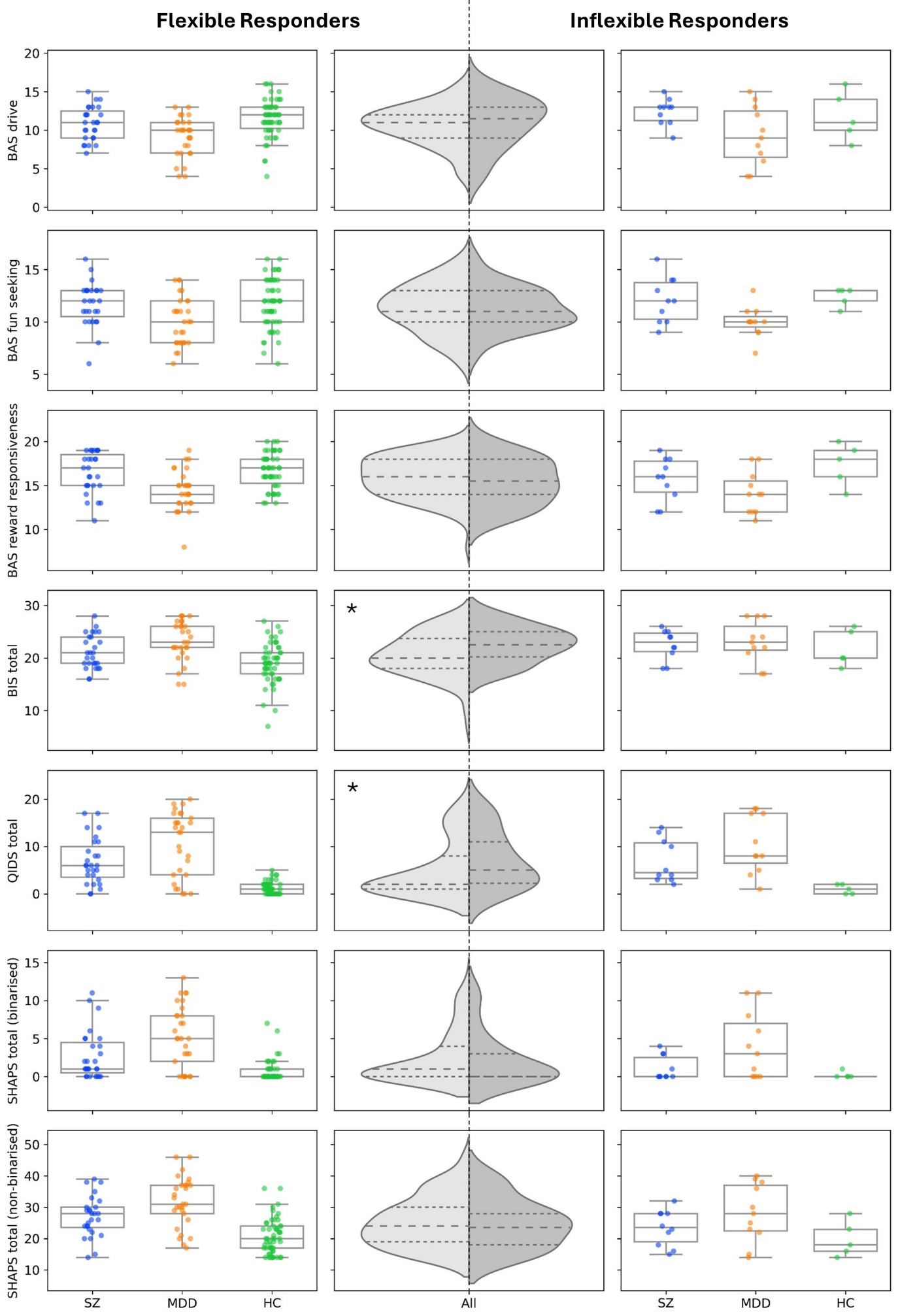


*Supplementary Figure 1 – Distributions of the questionnaire outcomes of the ‘flexible’ and inflexible responders. Violin plots show all participants, with dashed lines indicating quartiles, and boxplots are split by group. * p < .05 (for the difference between all flexible and inflexible responders).*

*Supplementary Figure 2*


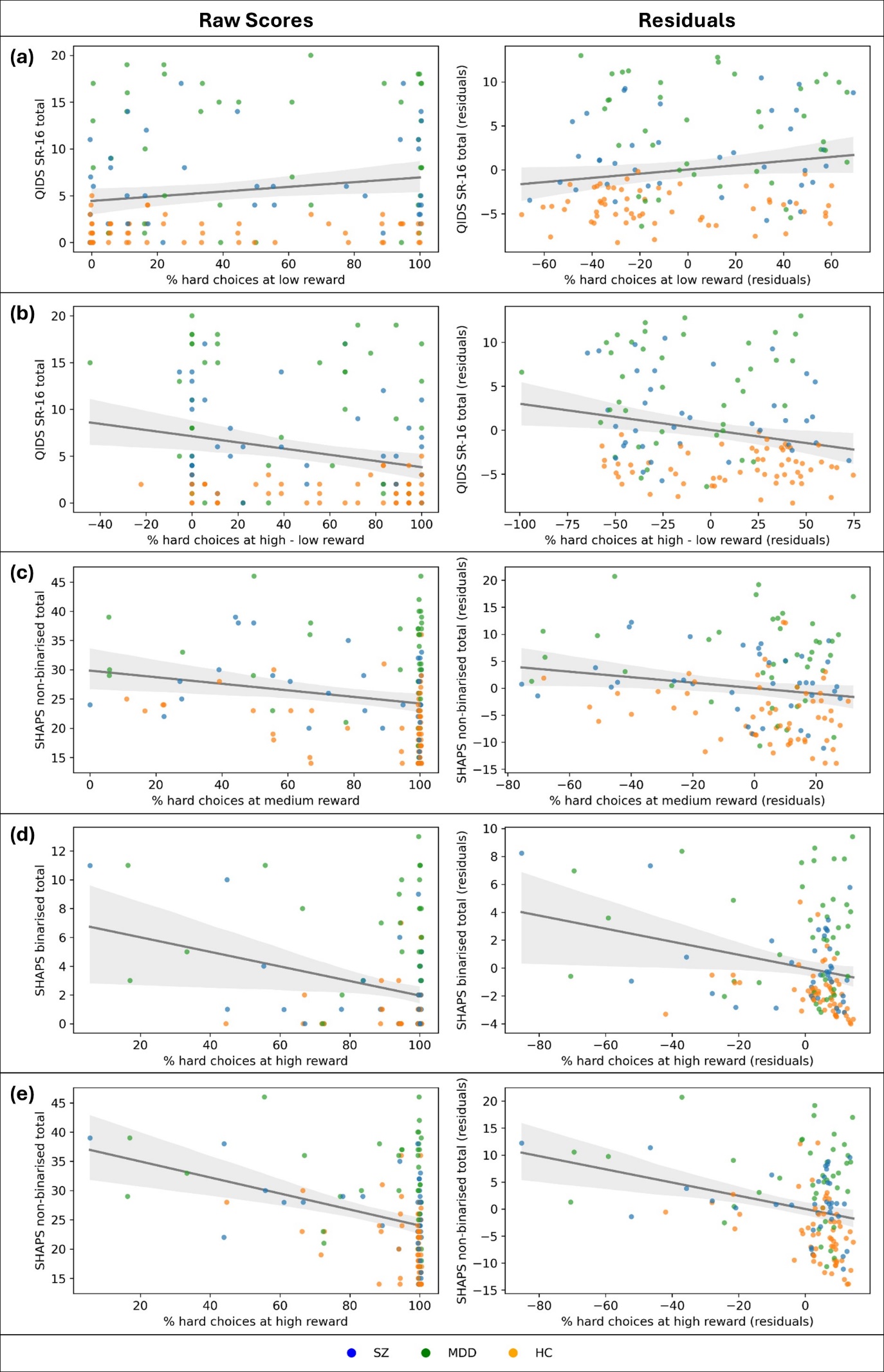


*Supplementary Figure 2 – Scatterplots of statistically significant Spearman’s partial correlations (covariates: site, sex, and age) between questionnaire and GSET outcomes (including inflexible responders). Left column shows the raw scores and right column shows the residuals of the outcomes after linear regression with the covariates. Across all participants, (a) r_S_(129)=0.22, p=.012, (b) r_S_(129)=-0.25, p=.004, (c) r_S_(129)=-0.21, p=.015, (d) r_S_(129)=-0.17, p=.047, (e) r_S_(129)=-0.29, p=.001.*

*Supplementary Figure 3*


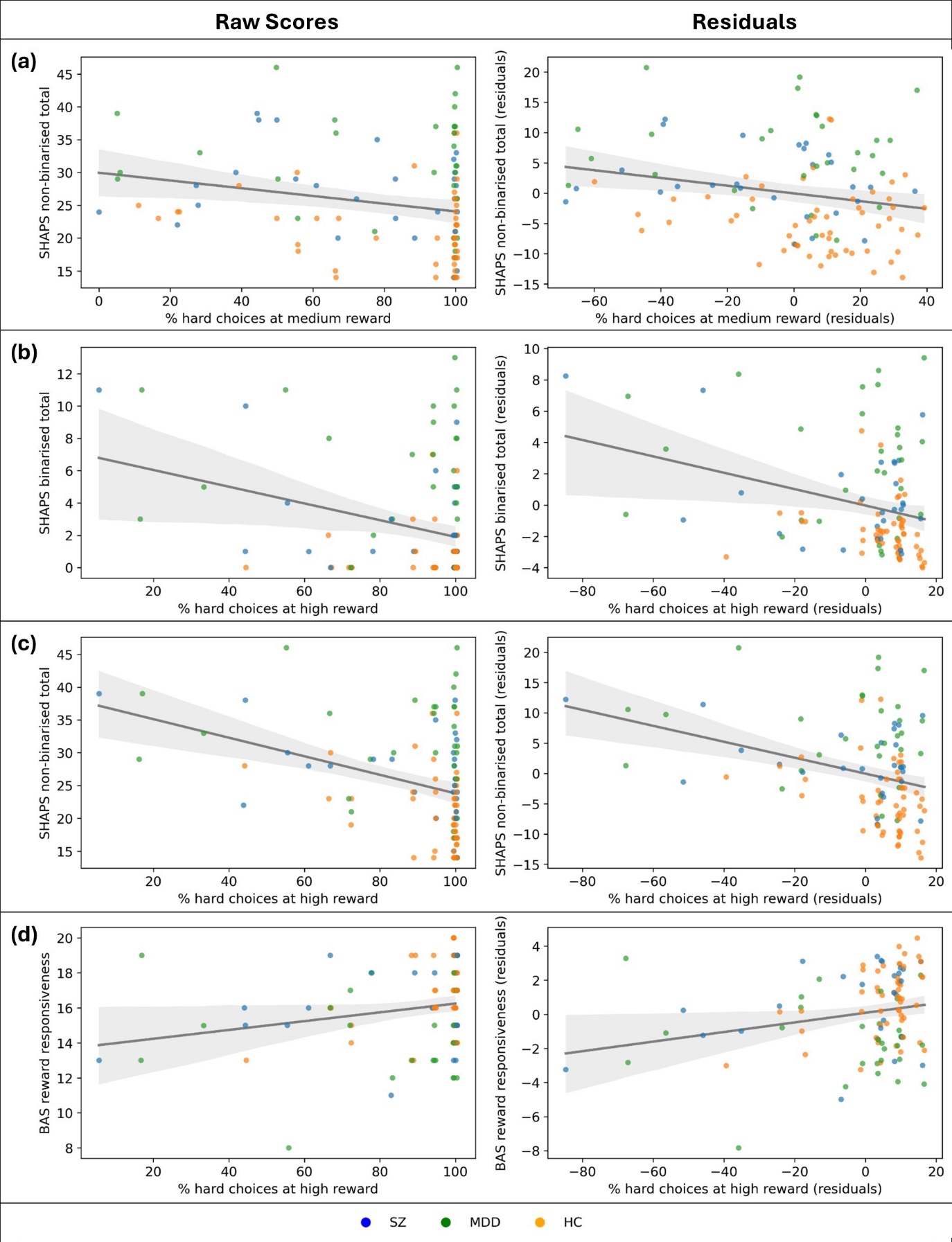


*Supplementary Figure 3 – Scatterplots of statistically significant Spearman’s partial correlations (covariates: site, sex, and age) between questionnaire and GSET outcomes (excluding inflexible responders). Left column shows the raw scores and right column shows the residuals of the outcomes after linear regression with the covariates. Across all participants, (a) r_S_(103)=-0.22, p=.023 (b) r_S_(103)=-0.25, p=.009, (c) r_S_(103)=-0.35, p<.001, (d) r_S_(103)=0.22, p=.022.*

*Supplementary Figure 4*


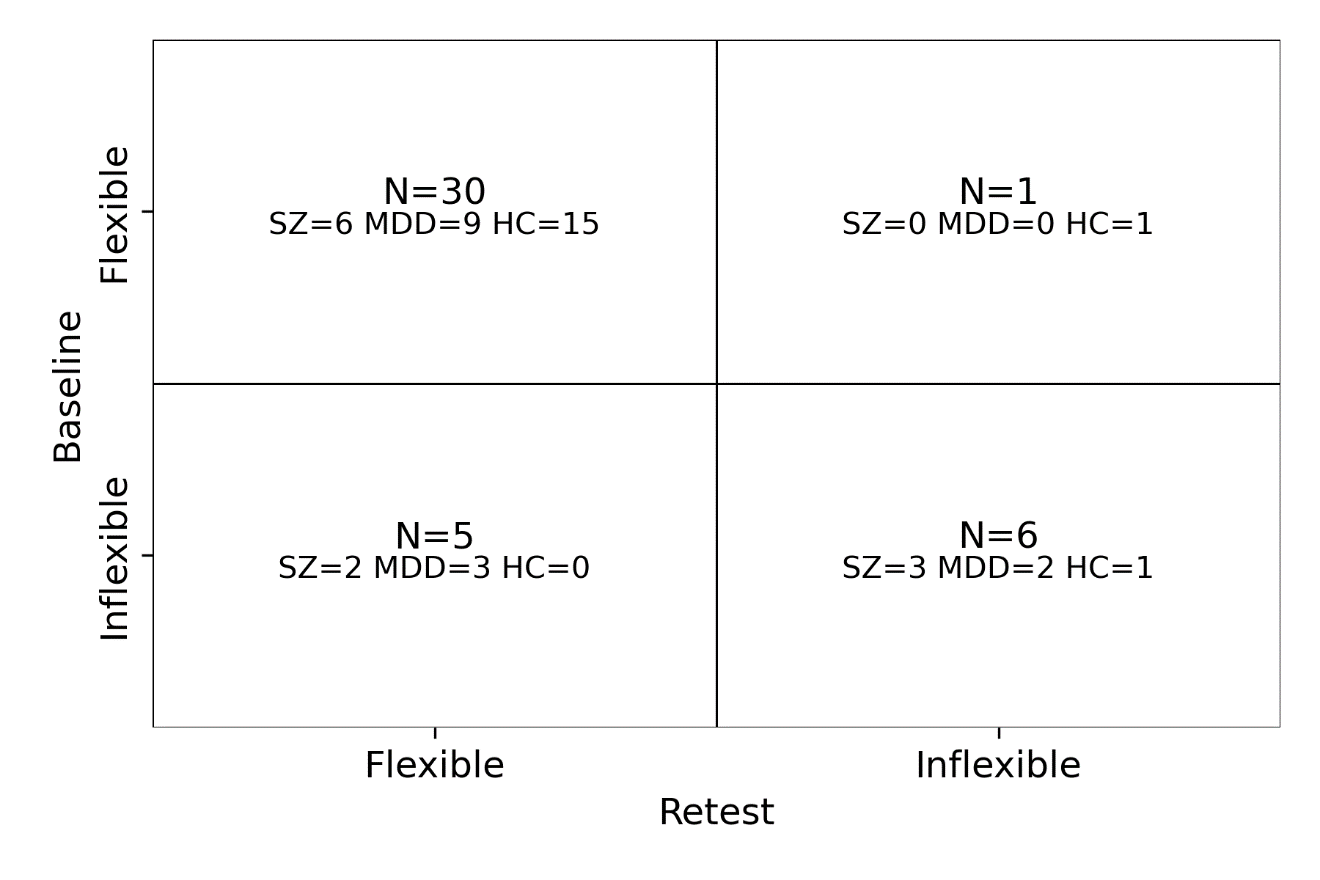


*Supplementary Figure 4 – Confusion matrix of the number of retest participants that were inflexible either at baseline and / or retest. Note: one participant only performed the GSET at retest and not at baseline and, therefore, is not counted here.*
